# The Effect of the Question Mark Option in Progress Testing: A Large-Scale Longitudinal Study

**DOI:** 10.5334/pme.1673

**Published:** 2025-12-03

**Authors:** Elise V. van Wijk, Jeroen Donkers, Peter C. J. de Laat, Ariadne A. Meiboom, Bram Jacobs, Jan Hindrik Ravesloot, René A. Tio, Frederike M. M. Oud, Jeroen P. Kooman, André J. A. Bremers, Alexandra M. J. Langers

**Affiliations:** 1 Center for Innovation in Medical Education, Leiden University Medical Center, The Netherlands; 2 School of Health Professions Education, Faculty of Health, Medicine and Life Sciences, Maastricht University, The Netherlands; 3 Department of Pediatrics, Erasmus Medical Center, Rotterdam, The Netherlands; 4 Department of General Practice and Elderly Care Medicine, Amsterdam University Medical Center, Amsterdam, The Netherlands; 5 Department of Neurology, University of Groningen, University Medical Center Groningen, Groningen, The Netherlands; 6 Department of Physiology, Amsterdam University Medical Center, Amsterdam, The Netherlands; 7 Department of Cardiology, Catharina Hospital Eindhoven, Eindhoven, The Netherlands; 8 Education Centre, Department of Medical Education, University Medical Center Utrecht, Utrecht, The Netherlands; 9 Department of Internal Medicine, Division of Nephrology, Maastricht University, The Netherlands; 10 Department of Surgery, Radboud University Medical Center, Nijmegen, The Netherlands; 11 Department of Gastroenterology and Hepatology, Leiden University Medical Center, Leiden, The Netherlands

## Abstract

**Introduction:**

Formula scoring, widely used in medical progress tests (PT), includes a question mark option to discourage guessing, but this feature may disadvantage risk-averse students and bias results due to test-taking strategies. To enhance reliability and more accurately assess ability, Dutch medical schools recently transitioned to a computer adaptive-PT (CA-PT) based on Item Response Theory, which adjusts question difficulty dynamically, excluding the question mark option. This provided a unique opportunity to evaluate the impact of the question mark option in a large cohort. We specifically explored the relationship between question mark use in conventional PT and performance on CA-PT.

**Methods:**

Retrospective data from medical students across seven faculties who took both PT formats were analyzed. Z-scores for total score and question mark score (number of unanswered questions) in the conventional PT, and theta score for the CA-PT were assessed. A linear model assessed the effect of the question mark score on theta, corrected for the conventional PT-score. Cluster analysis explored student subgroups per year.

**Results:**

Students with similar conventional PT scores who left more questions unanswered on the conventional PT generally performed better on CA-PT. This effect diminished as students advanced through their studies. Cluster analysis revealed a variable effect between different students, most pronounced in year 4, and a reverse effect in year 5.

**Discussion:**

Question mark option use significantly impacted student performance on PT, with a remarkable variability among students. This variability suggests that formula scoring captures more than knowledge alone, highlighting the need to align scoring methods with intended assessment goals.

## Introduction

In classical test theory (CTT), number-right scoring and formula scoring are the two primary scoring methods for calculating test scores on multiple-choice question (MCQ) assessments [1]. Number-right scoring awards points for correct answers without penalizing incorrect ones, while formula scoring deducts points for incorrect answers to discourage guessing. Additionally, formula scoring includes a question mark option, allowing students to acknowledge gaps in their knowledge without penalty [2]. This method has been widely used in medical progress tests (PT) across the Netherlands, Germany, Canada, and in the United Kingdom [3].

The rationale for adopting formula scoring in progress testing is twofold: to encourage students to reflect on their certainty and to provide an opportunity to indicate when they are unsure, thereby reducing guessing, particularly among early-stage students who are not yet expected to perform at an end-of-curriculum level [1,2,3,4,5]. From a decision theory perspective, formula scoring requires students to balance potential gains from answering against risks of penalties for incorrect responses. This trade-off reflects individual differences in risk aversion, a cognitive construct describing how people weigh uncertain outcomes [6,7]. More risk-averse students tend to select the question mark option to avoid penalties even when they possess partial knowledge, whereas more risk-taking students attempt more answers despite uncertainty [4,8,9]. As such, the question mark option may disadvantage risk-averse students, as they may score lower than peers with similar knowledge who take more risks [4,10,11,12].

Additionally, test-wiseness – the ability to improve performance through strategic use of the test format and management of uncertainty, independent of actual content mastery – may influence question mark use. It involves metacognitive awareness and test-taking strategies [13,14]. Therefore, question mark use may serve as a behavioural proxy, but it also introduces bias in score interpretation [1,4,15,16,17]. Ravesloot *et al*. demonstrated that formula scoring distorts knowledge measurement by mixing true ability with individual differences in the tendency to use the question mark option [4]. In their cohort, the question mark option accounted for 8% of the variance in formula scoring – a substantial effect that weakens construct validity.

Beyond individual test-taking behaviour, question mark option usage also depends on item-related factors. When clearly incorrect answers can be identified, students have a better chance of guessing correctly from the remaining options and scoring higher, rather than selecting the question mark, which yields 0 points. Moreover, gender differences in guessing behaviour further challenge the construct validity of test scores under formula scorning [4,11,18].

To improve the efficiency and reliability of the progress test (PT), the Dutch medical schools transitioned from a linear-fixed PT with formula scoring to a computer adaptive progress test (CA-PT) [19]. The CA-PT is based on Item Response Theory (IRT), which, unlike the CTT, does not assume that all items contribute equally to a student’s score. Instead, it uses item difficulty parameters of a set of calibrated questions to measure students’ abilities (‘theta’), without the need for a question mark option [20,21]. In the CA-PT, the difficulty level of the selected questions is adapted based on previous answers, providing a more accurate evaluation of student knowledge [20].

This transition to a computer-adaptive test provides a valuable opportunity to evaluate the impact of the question mark option in formula scoring on student performance within a large, summative, and longitudinal cohort of medical students at different educational stages. As students completed both PTs with a question mark option (conventional PT) and without it (CA-PT), this setting allows for a unique examination of how the question mark option influences students’ performance. These insights can guide the selection of formula scoring as scoring method. Therefore, the primary aim of this study was to explore the relationship between the question mark option in the conventional PT and student performance on the CA-PT. To establish the construct validity of this comparison, we first assessed the correlation between these formats over time. We hypothesized that the removal of the question mark option would have the greatest impact on the performance of junior students, as they tend to use the question mark option more frequently and have not yet learned how to use this option effectively. They may also be less convinced about their knowledge and therefore answer fewer questions. Their lack of confidence may lead them to answer fewer questions than optimal, potentially boosting their performance when the question mark option is removed.

## Methods

### Setting

In the Netherlands, eight universities offer medical education, each with a comparable curriculum structure comprising six years of undergraduate medical education. The curriculum is divided into a three-year (preclinical) Bachelor’s program followed by a three-year (clinical) Master’s program. The framework for undergraduate medical education defines the joint learning outcomes for both the preclinical and clinical phases, and is applicable to all medical students [22]. The preclinical phase primarily focuses on establishing a theoretical foundation and providing some essential basic skills, while the clinical phase is characterized by clinical rotations. The Dutch interuniversity medical PT is a longitudinal, comprehensive test that evaluates the development of students’ functional medical knowledge throughout the entire curriculum, benchmarking against peers at the same stage of study. There are four test administrations (i.e., test moments) for each of six academic years (September, December, February, and May) in which medical students of all eight Dutch medical schools participate. This results in 24 test moments for each student throughout the curriculum. As students progress through their academic years, the passing scores of the PT increase correspondingly. At the end of each academic year, the results from the four progress tests are combined into a summative decision (fail, pass, or good) [23]. To ensure content validity, the PT questions are administered according to a blueprint that prescribes the distribution of questions across relevant medical disciplines (***Supplemental Table 1***).

### Conventional progress test and formula scoring

The conventional PT was a linear-fixed test format, based on principles of the CTT [24]. It comprised 200 multiple-choice questions (MCQs), each with two to four answer options and a question mark option. Choosing the question mark option resulted in a neutral score of zero points, while selecting an incorrect answer incurred a penalty (negative score), and a correct answer earned one point. This formula scoring method was intended to discourage guessing. The total test score was calculated as the sum of item scores, expressed as a percentage of the maximum achievable score [2,19]. The MCQs were developed and reviewed by content experts, covering a broad range of medical knowledge domains.

### Computer adaptive progress test (CA-PT)

The CA-PT was introduced across all Dutch medical schools following our cross-over study in May 2022 [19]. Details of the transition between the test formats are described in that publication. The study results demonstrated a strong correlation between student performance on the conventional PT and CA-PT (r = 0.834), which supports the CA-PT as a valid and reliable measure of students’ knowledge level. Earlier research has also shown that CA-PT improves measurement precision and reliability across the entire ability spectrum compared to linear testing [25].

The CA-PT consists of 135 MCQs, of which 120 are calibrated, adaptive questions, and 15 are non-adaptive pretest questions used for calibration of future items. The adaptive algorithm selects questions from an item bank that, at the time of data collection, contained 5,613 calibrated questions with known item parameters.

The CA-PT applies an IRT framework to estimate each student’s ability (theta score) based on the pattern of correct and incorrect responses to the 120 calibrated items. The theta score, expressed on a continuous latent scale, is then transformed to the range of the conventional PT score scale to facilitate comparison with earlier PT results. The item bank is regularly reviewed and updated to maintain content coverage and psychometric quality [26].

### Participants

We used retrospective data from medical students at seven Dutch medical schools who participated in both the conventional PT and the CA-PT. Data from one medical school were excluded because the institution joined the PT after implementation of the CA-PT, resulting in a lack of conventional PT data.

### Study design and data collection

**Figure 1:**
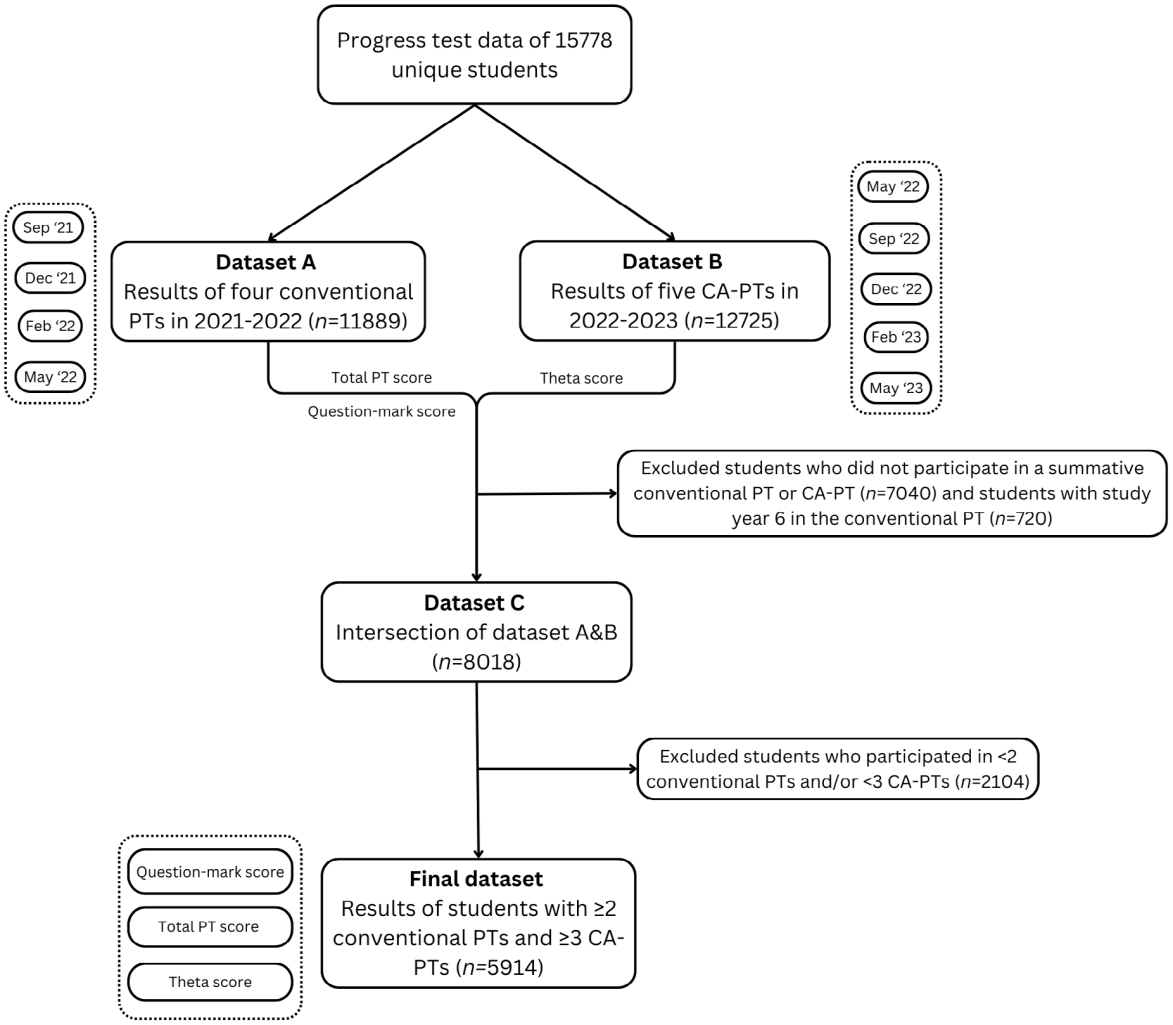
Flowchart of the data collection.

We used data from four conventional PTs administered between 2021 and 2022 (September 2021, December 2021, February 2022, and May 2022), and from five CA-PTs administered between 2022 and 2023 (May 2022, September 2022, December 2022, February 2023, and May 2023) (Figure 1). The PT results from May 2022 originate from our cross-over study [19], in which 1,432 students from three Dutch medical schools participated in both a conventional PT and CA-PT in the same time frame. For each PT, we extracted the test moment, medical school, and student ID. For the conventional PTs, we used the total PT score, and the “*question mark score*”. This question mark score captures the frequency of unanswered items as a discrete count (i.e., the total number of questions left unanswered by each student). For the CA-PTs, we used the theta score as the ability estimate. The theta score indicates the student’s ability as measured during the test. This continuous score takes into account the calibrated difficulty of the items answered correctly and incorrectly by the student. Due to COVID-19 restrictions in 2021–2022, some conventional PT-sessions were conducted online for part of the students and were non-proctored (*n* = 8,021/37,412 PTs in 2021–2022). These “*formative*” PT-sessions, which did not impact study credits due to the lack of supervision on the use of study materials, were excluded from our main analyses as previous findings indicated that their purely formative nature could affect test-taking motivation and student performance [27]. We performed a sensitivity analysis examining the impact of including versus excluding formative test results.

We linked the conventional PT data with the CA-PT data by student ID and selected data from students who participated in at least two conventional PTs and three CA-PTs (*n* = 5,914). We used a lower threshold for including the conventional PT (2 *vs*. 3), because we had to exclude the “*formative*” sessions, which would otherwise have limited the number of students eligible for this study. Each student was assigned a year group (1–6, representing the cohort) based on their earliest test moment in 2021–2022. Students in year group 6 were excluded due to the small, non-representative sample attending both the conventional PT and the CA-PT.

### Data analysis

We calculated average z-scores for both the total score and question mark score on the conventional PT, relative to all students in the same test moment group for each separate PT session. The number of tests per student can vary, since students can skip tests, which happened especially in the COVID-19 period. By taking the average z-scores per student, we implicitly imputed missing tests by person-means. In general, the z-score indicates the number of standard deviations a student’s score deviates from the mean of their group. It is calculated by subtracting the group mean from the individual score and dividing the result by the group standard deviation. Z-scores indicate the relative position of a student within their group and, in this study, were primarily used to correct for differences in difficulty between PTs, enabling comparison of results across tests.

In the conventional PT, consisting of 200 questions with an unknown difficulty level, the overall difficulty varies per test. Taking this into account, we computed z-scores per PT session per test moment group— one for the total score and one for the question mark score — yielding two z-scores per student per PT session. These were then averaged across all students within the same test moment group, resulting in one average z-score for the total score and one for the question mark score.

For the CA-PT, however, the difficulty level of all questions in the item bank is known. Therefore, no correction for test difficulty was needed here and we computed a z-score for the CA-PT theta score per test moment group, which was then averaged for each student, producing one average z-score for CA-PT theta per student. These three average z-scores per student (total score, question mark score, and theta score) were used in the analyses. We computed descriptive statistics for the selected student groups, including density plots to visualize the relationships between the different variables.

#### Correlation between conventional PT and CA-PT results

We computed the Pearson correlation coefficient to assess the convergent validity of the conventional PT and CA-PT by measuring the correlation between the total score on the conventional PT and the theta score on the CA-PT across multiple test sessions longitudinally.

#### Relationship between question mark option use and CA-PT performance

We investigated the relationship between the question mark score in the conventional PT and the theta score in the CA-PT using regression analysis and model-based clustering. Linear regression models were applied for each year group to correct for the conventional PT score and assess the effect of the question mark score on the theta score. Because we observed signs of underlying structure in the data, we decided to apply model-based clustering to further explore the subgroups in the year groups using the R package MClust, (version 5) [28]. This approach identified clusters of students with distinct patterns in their question mark and theta scores. To determine the number and type of clusters we used three methods: first the Bayesian Information Criterion (BIC) was computed for a range of model types and cluster counts (using mClustBIC, [29,30,31]), next Integrated Complete-data Likelihood (ICL), was computed for the same range (using mClustICL [29,30]). After visual inspection of both, Likelihood Ratio Tests (LRT) bootstrapping [29,30,32] was used on the best performing model type to obtain the optimal cluster count. Clusters were subsequently included as covariates in linear regression models to evaluate differences in behaviour between clusters within each year group. All statistical analyses were performed in R version 4.1.0 [29]. In the process, we performed several sensitivity analyses, including the impact of the exclusion of formative tests from the data (see ***Supplemental Report on Cluster Analysis*** for more details on the cluster analysis and sensitivity analyses).

To enhance comprehensibility, we provide suggestions regarding potential underlying student behaviours in selected clusters. These interpretations emerged from discussions among a subset of authors, and are informed by the results of the cluster analyses. While they are grounded in relevant literature, they cannot be directly validated with data from the present student cohort, as such information was not available. Accordingly, the term “*suggest*” is used when presenting these interpretations.

### Ethical approval

We used data from our cross-over study conducted in May 2022, for which ethical approval was granted by the Ethical Review Board of the Netherlands Association for Medical Education (NVMO) under reference NERB/2023.4.6. Participation in this CA-PT (May 2022) was voluntary, with students being informed beforehand and providing signed informed consent prior to its initiation. Retrospective data from other PT sessions were obtained from the PT database, which is maintained for the purposes of monitoring and improving PT administration. A waiver for the use of this retrospective data was granted by the NVMO Ethical Review Board. All data were pseudonymized before analysis.

## Results

### Descriptives

We included 5,914 students who participated in at least two summative conventional PTs and three CA-PTs for analysis (Figure 1). The number of students who participated in each possible combination of conventional PT and CA-PT sessions are shown in ***Supplemental Table 2***. In year group 3 we had only 415 students, because their participation in (CA-)PTs was reduced due to disruptions caused by the COVID-19 pandemic (i.e., they experienced a longer waiting period between the pre-clinical and clinical phase during which they did not participate in PTs). There were slight but statistically significant differences in the average z-scores per test moment in the bachelor phase of the selected students and the total student population. The differences ranged from –0.25 to +0.25, but were not systematic (***Supplemental Table 3***). ***Supplemental Table 4*** presents the mean absolute PT, question mark, and theta scores for each year group, to illustrate students’ test-taking behaviour across the year groups.

### Correlation between conventional PT and CA-PT results

We observed an overall Pearson correlation of 0.74 [95%CI: 0.72, 0.75] between the average z-score on the conventional PT and the CA-PT. The correlation was moderate to strong within each year group: Y1 (*n* = 1,067): 0.57 [95%CI: 0.52, 0.62]; Y2 (*n* = 1,017): 0.71 [95%CI: 0.67, 0.75]; Y3 (*n* = 415): 0.70 [95%CI: 0.63, 0.75]; Y4 (*n* = 2,615): 0.79 [95%CI: 0.77, 0.81]; Y5 (*n* = 800): 0.80 [95%CI: 0.77, 0.83]. All correlations were significant and had a p-value < 0.001.

### Relationship between question mark option use and CA-PT performance

**Figure 2:**
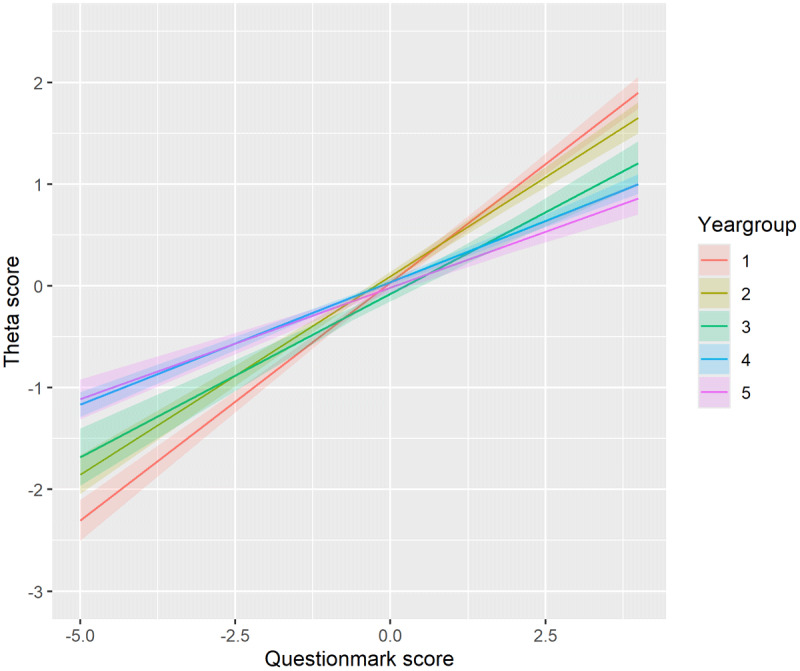
The effect of the question mark score (conventional PT) on the theta score (CA-PT), corrected for the PT score, for each year group. Positive theta scores indicate better-than-average performance, suggesting the individual is performing above the mean level. Negative theta scores suggest below-average performance, indicating the individual is performing below the mean level. The question mark score quantifies how frequently a student uses the “question mark” option. Higher scores indicate more frequent use while lower or negative scores indicate a preference for direct answers.

All results are presented as average z-scores. For simplicity and readability, however, we will refer to these values as “*scores*” throughout the remainder of this paper. Our linear model revealed a significant interaction between question mark score and theta score across all year groups. As illustrated in Figure 2, the question mark score (x-axis) positively affects the theta score (y-axis), after adjusting for the total score on the conventional PT (PT score). This positive effect suggests that students who left more questions unanswered on the conventional PT, indicating greater uncertainty, tended to perform better on the CA-PT for a given PT score. The positive effect was strongest in year group 1, but decreased as students progressed through their studies (Y1: 0.47; Y2: 0.39; Y3: 0.32; Y4: 0.24; Y5: 0.22). Our sensitivity analysis showed that including the formative tests in the data yielded similar results (***Supplemental Report on Cluster Analysis***).

**Figure 3:**
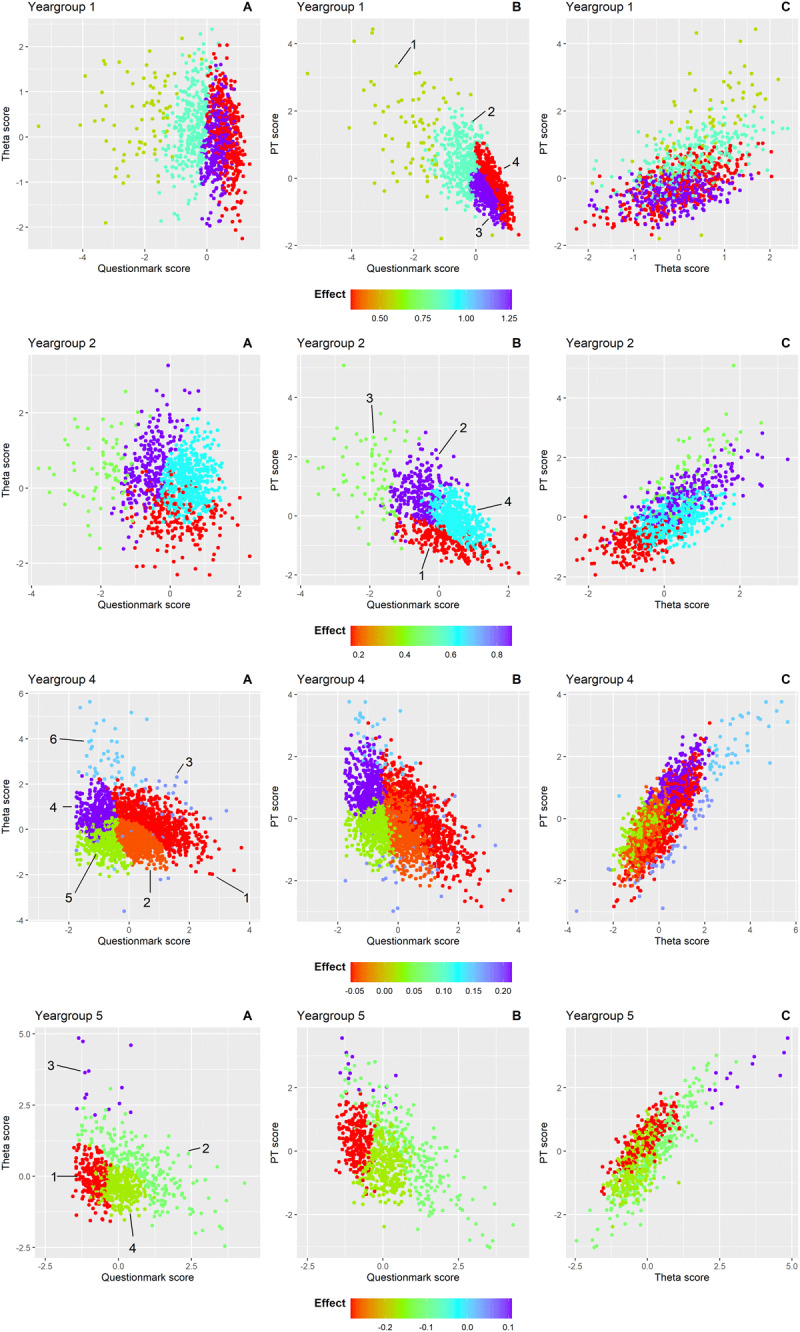
Clusters of students within each year group are shown in scatterplots from three perspectives to illustrate the relationship between question mark use (conventional PT) and theta score (CA-PT), adjusted for overall PT score: **A)** question mark score versus theta score **B)** question mark score versus PT score **C)** theta score versus PT score. The cluster numbers are indicated and encircled in that graph where the clusters are most distinctly separated for each year group. Each point represents an individual student, coloured according to the effect size of the question mark score on the theta score after adjusting for PT score within that cluster. Colours range from red (strong negative effect) to purple (strong positive effect). The color scale is consistent across all graphs within each year group but varies in range across groups to reflect differing effect sizes.

**Figure 4:**
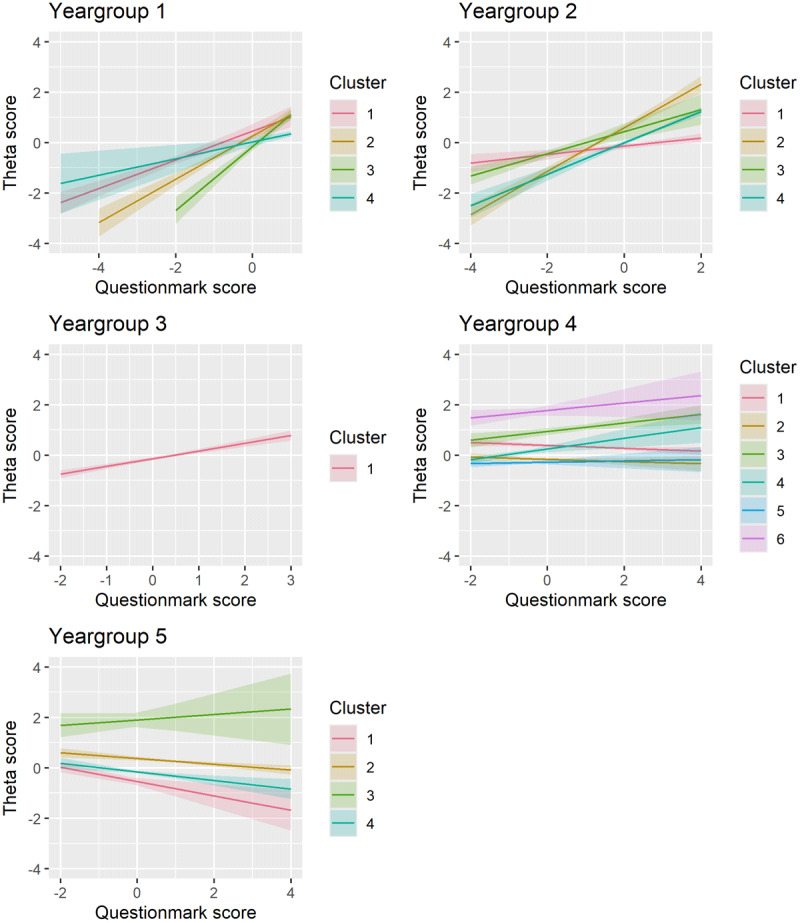
The effect of the question mark score (conventional PT) on the theta score (CA-PT), corrected for the PT score, within each cluster across the five year groups. Each cluster is indicated with a different colour. Optimal clustering solutions were identified using the Bayesian Information Criterion (BIC) and Integrated Complete-data Likelihood (ICL). BIC evaluates the model fit while penalizing complexity, ensuring an appropriate balance between accuracy and simplicity. ICL complements this by emphasizing well-separated and distinct clusters, reducing the risk of overfitting. Together, these criteria provided a framework to determine the number of clusters in each year group.

To examine the observed underlying structure in our data, we applied model-based clustering(see ***Supplemental Report on Cluster Analysis*** for more details). This analysis revealed the underlying structure of the effects across the year groups. Four clusters were identified in year groups 1, 2 and 5, while six clusters emerged in year group 4. Year group 3 did not exhibit distinct clustering. For each student, we assessed three variables: the CA-PT score, question mark score, and PT score. As our data is three-dimensional, the data are displayed in three separate projections to better visualize the cluster formations. Figure 3 presents the effect of question mark scores on theta scores within each cluster from the following perspectives: A) question mark score versus theta score; B) question mark score versus PT score; C) and theta score versus PT score. Each data point in the graph represents a student. For each student, we assessed three variables: the CA-PT score, question mark score, and PT score, which are displayed in three separate projections to better visualize the cluster formations. Within each cluster, we applied a linear model to examine the effect of question mark score on theta score, while adjusting for the PT score. The colour of each cluster indicates the effect size of this relationship. The shape and position of the clusters represent the type of students in each cluster based on their scores. For example, in cluster 3 of year group 1 (shown in purple), students had high question mark scores with a low variability (as seen in Figure 3 graph A and B), while their theta scores showed much greater variability (Figure 3 graph A). In this cluster, the linear model revealed a strong positive effect (indicated by purple datapoints) of question mark option usage on the theta score, after adjusting for PT score. These effects are also shown in Figure 4 by different line colours for each cluster.

Unlike Figure 2, which shows a consistent positive effect across all year groups, Figures 3 and 4 reveal a more nuanced pattern; positive effects of the question mark score on the theta score for the clusters in the first and second year group, but predominantly negative effects for the clusters in the fifth year group. This suggests that for less experienced students, greater reliance on the question mark option – leaving more questions unanswered – is associated with better performance on the CA-PT. Conversely, in more experienced students, higher use of the question mark option correlates with poorer CA-PT performance. Year group 4 showed the greatest variability in cluster effects (Figures 3 and 4). In the following paragraphs we will describe in more detail the clusters that stood out. Year group 3 was excluded from this analysis due to insufficient data for clustering. ***Supplemental Table 5*** shows the mean scores (PT, CA-PT, and question mark), and effect sizes for each cluster.

### Year group 1

In year group 1, students in ***cluster 3*** (*n* = 324) and ***cluster 4*** (*n* = 360) used the question mark option more than average in the conventional PT, with ***cluster 4*** students using it more often (mean [SD]: ***cluster 3:*** 0.34 [0.26]; ***cluster 4:*** 0.63 [0.29]). These clusters were difficult to separate clearly based on their question mark score and theta score, as shown by the overlap between clusters in Figure 3A. Their scores on the CA-PT were similar (***cluster 3:*** –0.13 [0.63]; ***cluster 4***: 0.03 [0.74]). However, their scores on the conventional PT differed with ***cluster 4*** students performing better (***cluster 3:*** –0.45 [0.36]; ***cluster 4:*** –0.21 [0.58]). The key difference was in how effectively they used the question mark option: in ***cluster 3***, abandoning the question mark option strongly boosted CA-PT performance as illustrated by the steepest line (light green) in Figure 4 (i.e., strong positive effect). In ***cluster 4***, the effect was much weaker (dark green line in Figure 4). This suggests that students in ***cluster 4*** were better at using the question mark option to improve their performance on the conventional PT compared to those in ***cluster 3***.

### Year group 2

Students in ***cluster 2*** (*n* = 243) used the question mark option below average (–0.58 [–0.44]), while achieving the highest mean CA-PT score (0.61 [–0.76]). Despite their limited use of the question mark option in relation to their peers, their CA-PT performance was boosted following the removal of the question mark option, as illustrated by the steep line in Figure 4 (i.e., strong positive effect). This suggests that they did not use the question mark option effectively, and the reliance on direct answers in the CA-PT resulted in a better performance. Similarly, students from ***cluster 4*** (*n* = 481) who exhibited the greatest uncertainty and the highest question mark usage (0.51 [0.41]) gained from the removal of the question mark option. These students also show a great improvement from a below-average PT score (–0.12 [0.47]) to an above-average CA-PT score (0.20 [0.53]). This suggests that while these students initially depended heavily on the question mark option, its removal encouraged more decisive responses, enhancing their CA-PT performance.

### Year group 4

In this year group, students in ***cluster 4*** (*n* = 411) answered most questions directly on the PT, reflected by the lowest question mark score (–0.99 [0.32]). Their strategic use of the question mark option resulted in the highest mean conventional PT score (1.02 [0.52]). The removal of the question mark option further boosted their CA-PT performance (0.68 [0.55]), reflected by the positive effect (Figure 4). In contrast, students in ***cluster 5*** (*n* = 518) experienced neither a benefit nor a disadvantage from the removal of the question mark option, as reflected by a near-neutral effect (horizontal slope of the blue line in Figure 4). This suggests that these students were well aware of their knowledge gaps and used the question mark option effectively.

### Year group 5

Students in ***cluster 1*** (*n* = 236) answered most questions directly on the conventional PT, reflected by the lowest question mark score (–0.85 [0.29]). They scored above average on the conventional PT (0.33 [0.61]), but their performance on the CA-PT was below average (–0.10 [0.57]). This pattern, together with the strong negative effect, suggests that these students used the question mark option strategically to improve their score on the conventional PT. However, without the question mark option on the CA-PT, their scores reveal a lower knowledge level that resulted in below-average CA-PT performance.

## Discussion

We found a strong correlation of average z-scores between the two PT formats over time, supporting their convergent validity and strengthening the justification for the switch to CA-PT [19]. This result allows score comparisons across the different PT formats, suggesting that observed score differences are primarily due to question mark usage. The overall effect of the question mark score on the theta score, adjusted for the PT score, was consistently positive across all year groups but diminished with student progression. While the general trend was positive, our cluster analysis exposed varying student behaviours within each year. Notably, year group 4 showed considerable variation in student behaviour, and in year group 5 the effect reversed, becoming predominantly negative.

The effect of question mark use on the theta score shifts notably over the curriculum, shifting from a strong positive effect in year group 1 to a predominantly negative effect in year group 5. This shift may reflect the development of students’ test-taking strategies and metacognitive regulation, as well as increased expectations and higher performance thresholds in later stages. Notably, the negative effects were evident at the cluster level, but not in the overall year 5 cohort. This discrepancy may reflect Simpson’s paradox, a statistical phenomenon in which a trend observed in several subgroups of data disappears or reverses when these groups are combined [33]. In our case, while individual clusters within year 5 showed a negative association between question mark use and theta score, the overall association for the full year 5 cohort appeared less pronounced. This can occur because the relative sizes or distributions of the clusters can change the weighted average of effects at the aggregated level, masking the underlying subgroup patterns. Recognizing this paradox highlights the importance of examining subgroup trends rather than relying solely on aggregated data.

Early in the curriculum, frequent question mark option use on the conventional PT correlated with higher CA-PT scores, potentially indicating more risk-averse behaviour or limited self-confidence, consistent with emerging metacognitive skills and self-efficacy development [1,4,34]. Students who answered fewer questions on the conventional PT, but performed well, may have been more risk-averse and thus benefitted from the mandatory answering format of the CA-PT [2,11,12]. Conversely, students who took more risks by answering more questions despite uncertainty performed better on the conventional PT, but were disadvantaged by the lack of a question mark option in the CA-PT. These behavioral differences, shaped by individual risk tolerance, self-monitoring, and test-wiseness, may lead to similar scores on the conventional PT, but reflect distinct underlying constructs. We emphasize that these behavioural interpretations are consistent with prior research, but lack direct empirical support in the present study.

In later years, however, the association between question mark use and performance reversed. Students answering fewer questions on the conventional PT tended to perform worse on the CA-PT, suggesting that effective use of the question mark option may have masked limited knowledge. This shift could reflect either metacognitive awareness or the development of test-taking strategies. As shown by Cecilio-Fernandes *et al*. [35], it is more likely that senior students had developed refined strategic approaches rather than improved their metacognitive accuracy. Their study observed a decline in students’ judgment accuracy over time. While metacognitive theory generally associates increased knowledge with improved self-monitoring [36,37,38], increasing clinical experience and higher performance expectations may instead lead students to adopt alternative strategies or become overconfident [39,40]. Additionally, the perceived cost-benefit balance of guessing versus omitting may change, particularly if students consider the penalty for incorrect answers to be low [41].

The lack of a consistent pattern in year group 4 suggests a heterogenous group of students with varying behaviours. While the overall effect remained predominantly positive, we also observed clusters with a near-neutral effect. This variation may reflect different levels of test-wiseness (e.g., effective question mark option use), self-assessment, and different prior trajectories in this year group [13,35]. Some students in year 4 transitioned directly from year 3, while others completed a research internship or pursued other activities before starting clinical rotations, causing heterogeneity among the students in this year group. Finally, students who tended to answer more questions on the conventional PT generally achieved higher scores on both PT formats. This effect was most pronounced in the relatively small, but clearly distinguished clusters of best-performing students, whose scores were less affected by question mark usage (e.g., cluster 3 in year group 2). Overall, the wide diversity in student behaviour observed across and within year groups in our study suggests that formula scoring assesses constructs beyond knowledge level, including metacognitive awareness, test strategies, and risk-tendencies.

## Strengths and limitations

The strengths of this study include its multi-center design, the large cohort of medical students at different stages in the medical curriculum, and the use of summative PT results, minimizing selection bias regarding student participation. The longitudinal design provided a nuanced understanding of formula scoring effects on student performance.

This study also faced limitations, including potential selection bias favoring higher-performing students. In general, there is a difference in PT performance between the different medical schools. Due to variation in COVID-19 testing policies between the medical schools, particularly in years 1–3 where some of the schools introduced unsupervised formative PTs, a selection occurred after excluding the students from schools that scheduled formative PTs at certain time points. This explains the slight but statistically significant differences in z-scores compared to the total population. Our sensitivity analysis including both formative and summative tests showed no fundamental change in the overall patterns or conclusions. Additionally, lack of access to student characteristics hindered a more in depth-analysis of the underlying mechanisms or traits driving the observed student behaviour. Consequently, our interpretation of the underlying behaviour explaining the observed cluster scores and effects are speculative and based on earlier research. The differences in test formats of the conventional PT and the CA-PT (flexible navigation through the questions *vs*. direct answering format) may have influenced student strategies and performance, complicating direct comparisons. Cluster analysis sensitivity to input data, outliers, and nondeterminism, may have influenced the clusters identified, particularly where overlapping clusters with similar scores exhibited different effects. While this may have affected individual cluster assignments, we anticipate that it did not significantly impact the overall observed group-level patterns. However, some clusters exhibited large score variances, making it difficult to draw definitive conclusions. Although this was a national multicenter study including institutions with different educational cultures, all participating schools were based in the Netherlands. Validation of these findings in other educational systems and cultural contexts would therefore be of interest.

## Implications and future research

Our results support and expand on prior research that formula scoring affects construct validity of test scores [4,11]. The high variability in question mark use and its impact on performance suggest that formula scoring introduces bias, potentially distorting the measurement of students’ knowledge. This raises concerns about its continued use in progress testing, particularly given its inconsistent impact on subgroups of students with similar ability levels.

These findings underscore the importance of aligning scoring methods with the intended purpose of the assessment. If the goal is to assess student knowledge development over time, removing formula scoring may yield more valid scores. If, on the other hand, fostering metacognitive skills such as self-monitoring and risk assessment is also a goal [42], formula scoring or alternative methods that take into account metacognitive skills, like certainty-based marking (CBM) [43], may be more appropriate to capture these constructs.

CBM, which allows students to indicate their level of confidence in each answer and adjusts scoring accordingly [43], has been shown in previous studies to improve the accuracy of knowledge assessment and self-reflection [44,45]. In particular, CBM can provide richer diagnostic information by distinguishing between lack of knowledge and overconfidence, which formula scoring does not explicitly capture. It has been associated with increased reliability and better discrimination of lower-performing students’ knowledge [46], and students generally perceive it as fair and helpful for focusing their learning [47]. Nevertheless, individual differences in risk behaviour can affect confidence reporting, with risk-averse students tending to understate certainty on high probability items, highlighting the need for appropriate training and careful interpretation [48].

At the policy level, national test committees should critically evaluate whether formula scoring aligns with the overarching purpose of progress testing. In assessments where formula scoring is considered the best option, supportive interventions to mitigate potential disadvantages for affected students are essential to ensure fairness and validity. Such interventions could include targeted training in test-taking and metacognitive strategies, clearer guidance on how the scoring system operates, and ongoing monitoring to identify and support students at risk of underperformance due to their test-taking behaviour.

Finally, our findings also suggest that further qualitative research is warranted to understand theunderlying traits or mechanisms that drive differences in test-taking behaviour across student clusters. Interviews, focus groups or a retrospective think-aloud protocol with students could provide insights into how they perceive and respond to the different scoring methods, which could inform the design of assessments.

## Conclusion

Our study demonstrates that question mark option use in formula scoring significantly influences student performance on the PT, with the effect varying across different stages of the curriculum. The great variability suggests that formula scoring measures not only knowledge, but also other student constructs, potentially introducing biases. Careful consideration of scoring methods aligned with the assessment goals is essential to ensure valid and reliable test outcomes.

## Data availability statement

The data is available upon request.

## Additional Files

The additional files for this article can be found as follows:

10.5334/pme.1673.s1 Supplemental Table 1. Blueprint of PT.

10.5334/pme.1673.s2 Supplemental Table 2. Number of students in PT and CA-PT.

10.5334/pme.1673.s3 Supplemental Table 3. Score differences per test moment.

10.5334/pme.1673.s4 Supplemental Table 4. Mean raw scores of theta, PT score and question mark score.

10.5334/pme.1673.s5 Supplemental Table 5. Mean z-scores for each cluster.

10.5334/pme.1673.s6 Supplemental Report on Cluster analysis. Determining the model and number of clusters.
