# Supplementary material for: The Effect of the Question Mark Option in Progress Testing: A Large-Scale Longitudinal Study
Source: Perspect Med Educ. 2025 Dec 3;14(1):891–904. doi: 10.5334/pme.1673 (PMC12680002; doi:10.5334/pme.1673)
Supplement: Supplemental Table 1. — Blueprint of PT. [file pme-14-1-1673-s1.pdf]

Supplemental Table 1. Blueprint used in the conventional progress test (**A**) and in the (**B**) computer adaptive progress test.

A.

**Discipline**	**Number of questions**
Anatomy	13
Biochemistry/Genetics/Histology/Molecular Cell Biology	18
Surgery	17
Dermatology/Ear. Nose. Throat/Ophthalmology	14
Epidemiology/Statistics	8
Pharmacology	9
Physiology	11
Geriatrics	8
Gynecology/Obstetrics	7
General Practice	20
Internal Medicine	26
Pediatrics	12
Metamedics	5
Neurology	7
Pathology/Immunology/Microbiology	10
Psychiatry/Psychology	12
Social Medicine	3
Total	200

B.

**Discipline**	**Number of questions**
Anatomy	7-8
Biochemistry/Genetics/Histology/Molecular Cell Biology	10-11
Surgery	10-11
Dermatology/Ear. Nose. Throat/Ophthalmology	8-9
Epidemiology/Statistics	4-5
Pharmacology	5-6
Physiology	6-7
Geriatrics	4-5
Gynecology/Obstetrics	4-5
General Practice	12
Internal Medicine	15-16
Pediatrics	7-8
Metamedics	3
Neurology	4-5
Pathology/Immunology/Microbiology	6
Psychiatry/Psychology	7-8
Social Medicine	1-2
Total	120
