# Supplementary material for: The Effect of the Question Mark Option in Progress Testing: A Large-Scale Longitudinal Study
Source: Perspect Med Educ. 2025 Dec 3;14(1):891–904. doi: 10.5334/pme.1673 (PMC12680002; doi:10.5334/pme.1673)
Supplement: Supplemental Table 2. — Number of students in PT and CA-PT. [file pme-14-1-1673-s2.pdf]

Supplemental Table 2. Number of students who completed each combination of the conventional progress and computer-adaptive progress test.

**Conventional progress test**	**Computer adaptive progress test**				
	**1**	**2**	**3**	**4**	**5**
**1**	48	141	216	374	45
**2**	145	176	389	950	190
**3**	197	338	827	2115	365
**4**	240	184	253	655	170

Row labels (1-4) indicate how many conventional PT sessions a student completed; column labels (1-5) indicate how many CA-PT sessions a student completed. The value in each cell represents the number of students who participated in that combination of sessions. Students highlighted in yellow were included in our analyses.
