# Supplementary material for: The Effect of the Question Mark Option in Progress Testing: A Large-Scale Longitudinal Study
Source: Perspect Med Educ. 2025 Dec 3;14(1):891–904. doi: 10.5334/pme.1673 (PMC12680002; doi:10.5334/pme.1673)
Supplement: Supplemental Table 3. — Score differences per test moment. [file pme-14-1-1673-s3.pdf]

Supplemental Table 3. The differences in average z-scores for the theta-score (CA-PT), PT-score (conventional PT), and question mark score between our study population and the entire student population expressed as p-values of independent t-tests.

Year	Test moment	Theta score	PT score	Question mark
1	1	*NA*[a]	**0.000**	**0.000**
	2	*NA*	0.505	0.825
	3	*NA*	0.156	**0.000**
	4	*NA*	0.061	0.274
2	5	**0.018**	0.865	**0.000**
	6	0.640	0.463	0.964
	7	**0.013**	0.319	**0.033**
	8	0.062	0.370	0.729
3	9	**0.000**	**0.010**	0.944
	10	0.825	0.283	**0.005**
	11	**0.000**	**0.000**	0.038
	12	0.792	0.141	0.305
4	13	0.057	0.663	0.948
	14	0.923	0.297	0.437
	15	0.239	0.388	0.365
	16	0.824	0.850	0.649
5	17	0.277	0.490	0.700
	18	0.470	0.949	0.897
	19	0.726	0.638	0.526
	20	0.744	0.721	0.459
6	21	0.476	*NA*	*NA*
	22	0.467	*NA*	*NA*
	23	0.476	*NA*	*NA*
	24	0.259	*NA*	*NA*

[a] Not applicable due to small samples. P-values <0.05 are presented in bold and highlighted in yellow boxes
