# Supplementary material for: The Effect of the Question Mark Option in Progress Testing: A Large-Scale Longitudinal Study
Source: Perspect Med Educ. 2025 Dec 3;14(1):891–904. doi: 10.5334/pme.1673 (PMC12680002; doi:10.5334/pme.1673)
Supplement: Supplemental Table 4. — Mean raw scores of theta, PT score and question mark score. [file pme-14-1-1673-s4.pdf]

Supplemental Table 4. Mean raw scores on the CA-PT (theta score), conventional PT (PT score), and question mark option (question mark score).

**Year group**	**Theta score**[a]	**PT score**	**Question mark score**
1 (*n*=1067)	-0.16 (0.20)	10.41 (3.90)	76.03 (11.05)
2 (*n*=1017)	0.04 (0.20)	20.39 (5.85)	54.45 (12.56)
3 (*n*=415)	0.11 (0.20)	30.04 (8.10)	33.59 (13.69)
4 (*n*=2615)	0.29 (0.19)	38.95 (8.87)	20.80 (11.34)
5 (*n*=800)	0.33 (0.17)	44.26 (8.61)	14.27 (9.22)

[a]All scores are expressed as mean (standard deviation). PT-score and question-mark score are on a scale of 0-100. Theta scores (CA-PT) are on a logit scale.
