# Supplementary material for: The Effect of the Question Mark Option in Progress Testing: A Large-Scale Longitudinal Study
Source: Perspect Med Educ. 2025 Dec 3;14(1):891–904. doi: 10.5334/pme.1673 (PMC12680002; doi:10.5334/pme.1673)
Supplement: Supplemental Table 5. — Mean z-scores for each cluster. [file pme-14-1-1673-s5.pdf]

Supplemental Table 5. Mean z-scores on the CA-PT (theta score), conventional PT (PT score), question mark option (question mark score) and the effect size for each cluster across the five year groups.

**Year group**	**Cluster**	**Theta score**[a]	**PT score**	**Question mark score**	**Effect**
1	1 (*n*=74)	0.47 (0.82)	1.48 (1.26)	-2.06 (0.94)	0.57
	2 (*n*=309)	0.24 (0.75)	0.50 (0.64)	-0.46 (0.38)	0.86
	3 (*n*=324)	-0.13 (0.63)	-0.45 (0.36)	0.34 (0.26)	1.26
	4 (*n*=360)	0.03 (0.74)	-0.21 (0.58)	0.63 (0.29)	0.33
2	1 (*n*=221)	-0.76 (0.54)	-0.86 (0.38)	0.20 (0.70)	0.17
	2 (*n*=243)	0.61 (0.76)	0.74 (0.61)	-0.58 (0.44)	0.86
	3 (*n*=72)	0.53 (0.80)	1.33 (1.16)	-1.95 (0.67)	0.44
	4 (*n*=481)	0.20 (0.53)	-0.12 (0.47)	0.51 (0.41)	0.62
3	1 (*n*=415)	-0.10 (0.87)	-0.06 (0.89)	0.13 (0.90)	0.31
4	1 (*n*=708)	0.33 (0.73)	-0.02 (0.94)	0.86 (0.76)	-0.06
	2 (*n*=875)	-0.39 (0.45)	-0.33 (0.63)	0.19 (0.37)	-0.04
	3 (*n*=57)	0.49 (1.17)	-0.85 (0.91)	0.54 (1.00)	0.17
	4 (*n*=411)	0.68 (0.55)	1.02 (0.52)	-0.99 (0.32)	0.21
	5 (*n*=518)	-0.48 (0.52)	-0.28 (0.46)	-0.73 (0.33)	0.03
	6 (*n*=46)	3.11 (1.21)	2.25 (0.80)	-0.66 (0.57)	0.15
5	1 (*n*=236)	-0.10 (0.57)	0.33 (0.61)	-0.85 (0.29)	-0.28
	2 (*n*=231)	0.30 (0.93)	0.01 (1.23)	0.89 (1.06)	-0.11
	3 (*n*=13)	3.22 (0.98)	2.36 (0.63)	-0.65 (0.69)	0.11
	4 (*n*=320)	-0.39 (0.40)	-0.42 (0.62)	0.05 (0.41)	-0.17

[a]All scores are expressed as mean (standard deviation). PT: (conventional) progress test.
