# Supplementary material for: The Effect of the Question Mark Option in Progress Testing: A Large-Scale Longitudinal Study
Source: Perspect Med Educ. 2025 Dec 3;14(1):891–904. doi: 10.5334/pme.1673 (PMC12680002; doi:10.5334/pme.1673)
Supplement: Supplemental Report on Cluster analysis. — Determining the model and number of clusters. [file pme-14-1-1673-s6.pdf]

Supplemental Report on Cluster analysis

This document contains the details of the cluster analyses, its sensitivity analyses and the determination of effects per cluster. The document also includes a sensitivity analysis regarding the exclusion of formative tests from the data, both for the clusters and for their effects.

Determining the model and number of clusters

All cluster analyses have been done using package MClust for mixture model clustering [1] in R [2].

Step 1: Model selection

BIC- and ICL-plots per year group ("jaargroep") for 1-9 clusters and all models, using mClustBIC en mClustICL:

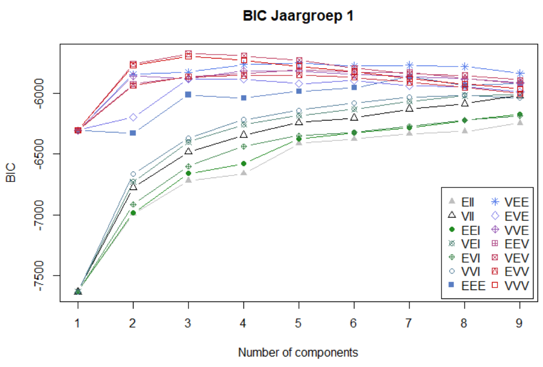

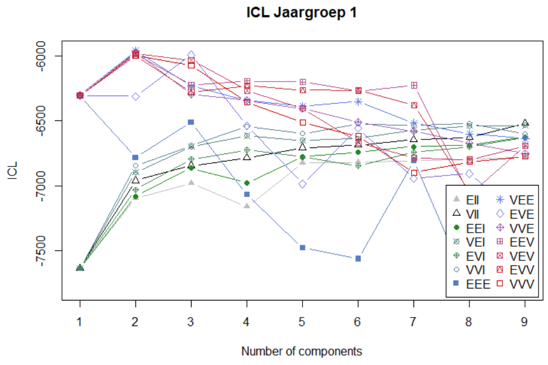

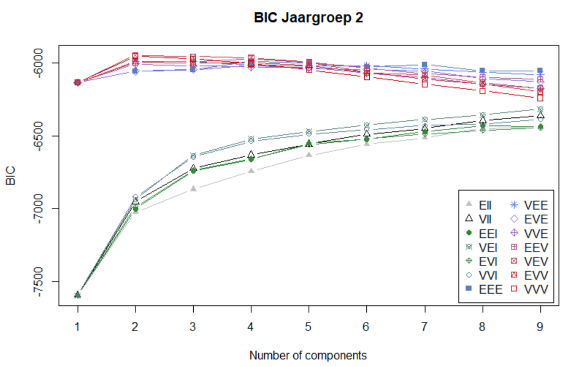

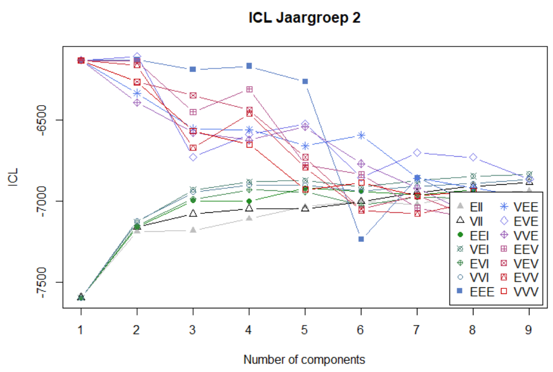

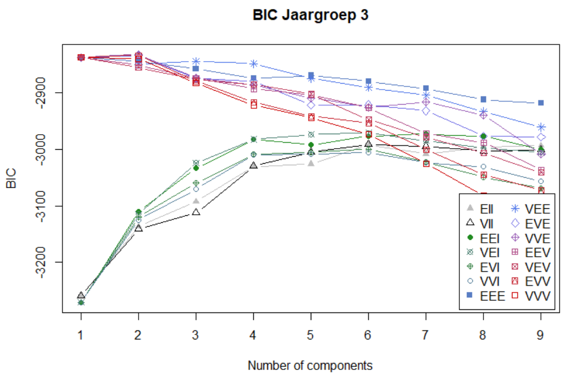

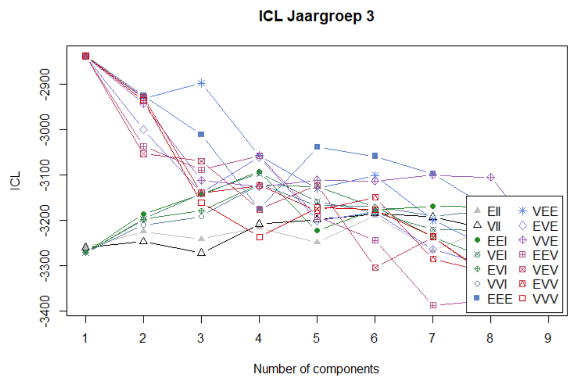

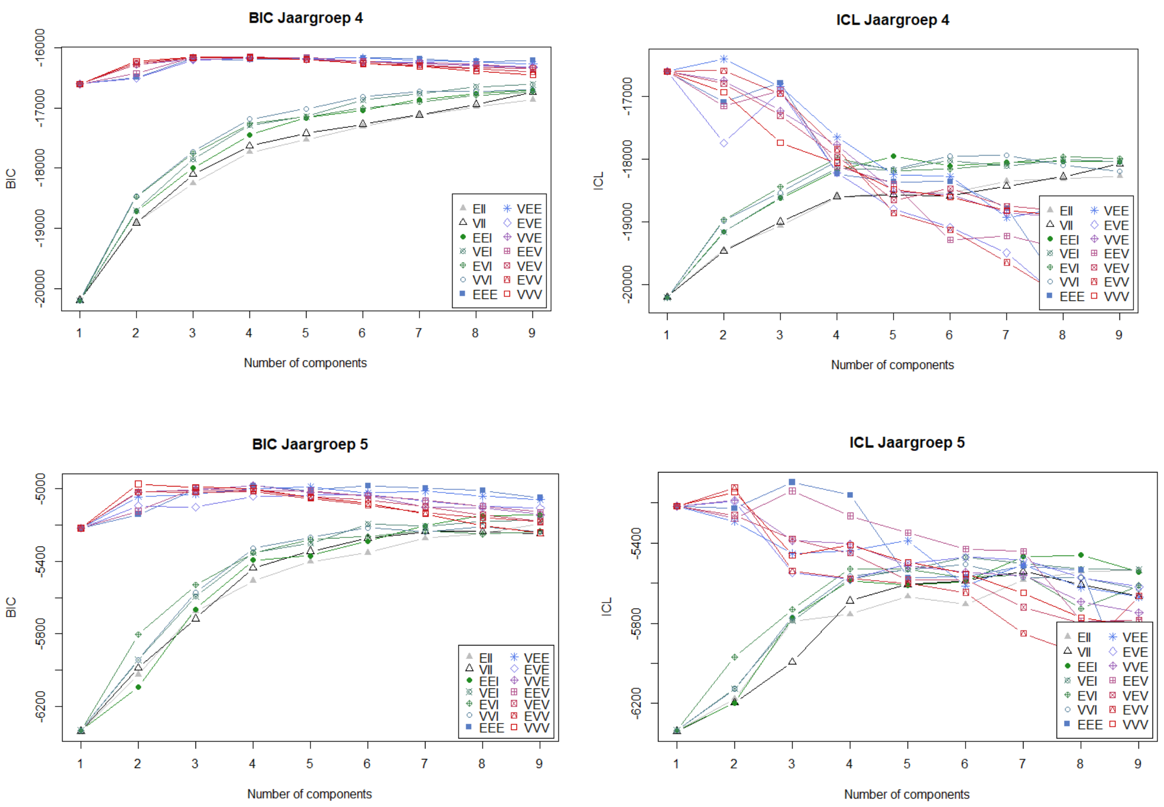

In general, the ICL-measure is more conservative in the number of clusters then the BIC-measure. Model VVV is (close to) optimal in all year groups. Since this model (varying volume, shape and orientation) puts the least restrictions on the cluster shapes, and we do not have theoretical reasons to limit the cluster shapes, the VVV model is preferred.

Step 2: Number of clusters

To determine the optimal number of clusters, we apply a sensitivity analysis using bootstrapping. For increasing number of clusters, clustering is repeated many times for a slightly altered dataset, then a likeliratio test is performed per transition.

From the documentation of Mclust:

The implemented algorithm for computing the LRT observed significance using the bootstrap is the following. Let G_0 be the number of mixture components under the null hypothesis versus G_1=G_0+1 under the alternative. Bootstrap samples are drawn by simulating data under the null hypothesis. Then, the p-value may be approximated using eq. (13) on McLachlan and Rathnayake (2014). Equivalently, using the notation of Davison and Hinkley (1997) it may be computed as

$$\text{p-value} = \frac{1+\#\{LRT_b^* \geq LRTS_{obs}\}}{B+1}$$

where
B = number of bootstrap samples

$LRTS_{obs}$ = LRTS computed on the observed data
LRT_{b^*} = LRTS computed on the *b*-th bootstrap sample.

The procedure stops when the p value exceeds 0.05.

Output of the bootstrapping Likelihood Ratio Test for model VVV with 999 replications for each of the year groups:

Year group 1:

	LRTS	bootstrap p-value
1 vs 2	607.64257	0.001
2 vs 3	142.95388	0.001
3 vs 4	37.40394	0.002
4 vs 5	20.80377	0.162

➔ *4 clusters.*

Year group 2:

	LRTS	bootstrap p-value
1 vs 2	249.94996	0.001
2 vs 3	50.35907	0.001
3 vs 4	37.43141	0.006
4 vs 5	26.56939	0.062

➔ *4 clusters.*

Year group 3:

	LRTS	bootstrap p-value
1 vs 2	57.85704	0.001
2 vs 3	17.76919	0.225

➔ *1 cluster.*

Year group 4:

	LRTS	bootstrap p-value
1 vs 2	459.10377	0.001
2 vs 3	144.17910	0.001

3 vs 4	73.83224	0.001
4 vs 5	44.68811	0.001
5 vs 6	17.52316	0.191

➔ *5 clusters.*

Year group 5:

	LRTS	bootstrap p-value
1 vs 2	311.50921	0.001
2 vs 3	48.64174	0.001
3 vs 4	58.90640	0.002
4 vs 5	18.48973	0.313

➔ *4 clusters.*

Step 3: Clustering per year group

Below we provide the outcome of the clustering with the above determined amount of clusters. In the figures and results below, variable *avztheta* is the average z-score for the CA-PT score theta, *avzvgtscore* is the average z-score for the PT score, and *avzvgtscore* is the average z-score for the question mark score in the PT.

Year group 1:

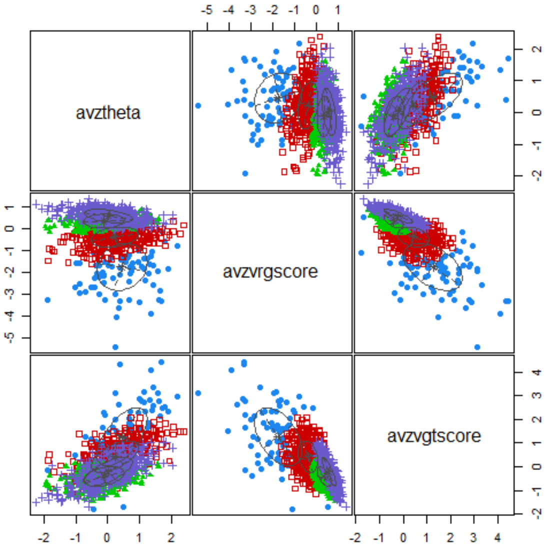


```
----------------------------------------------------
Gaussian finite mixture model fitted by EM algorithm
----------------------------------------------------

Mclust VVV (ellipsoidal, varying volume, shape, and orientation) model with 4 components:

 log-likelihood    n df       BIC       ICL
      -2726.519 1067 39 -5724.969 -6356.94

Clustering table:
  1   2   3   4
 74 309 324 360

Mixing probabilities:
         1          2          3          4
0.08439164 0.29583188 0.31911198 0.30066450

Means:
                  [,1]       [,2]       [,3]        [,4]
avztheta     0.4527673  0.2239285 -0.1206342  0.02534001
avzvrgscore -1.7480717 -0.3739189  0.3656247  0.59249683
avzvgtscore  1.2608522  0.4269417 -0.4151802 -0.20594912

Variances:
[,,1]
             avztheta avzvrgscore avzvgtscore
avztheta    0.6187844   0.1383193   0.5948008
avzvrgscore 0.1383193   1.1708377  -0.6237662
avzvgtscore 0.5948008  -0.6237662   1.5200098
[,,2]
              avztheta avzvrgscore avzvgtscore
avztheta    0.54979117  0.09619871  0.28935360
```

```
avzvrgscore 0.09619871  0.21172571 -0.08100543
avzvgtscore 0.28935360 -0.08100543  0.44053848
[,,3]
            avztheta avzvrgscore avzvgtscore
avztheta    0.40870731  0.01496014  0.10106559
avzvrgscore 0.01496014  0.10262184 -0.08143467
avzvgtscore 0.10106559 -0.08143467  0.17129866
[,,4]
            avztheta avzvrgscore avzvgtscore
avztheta     0.5652455 -0.10132574   0.3027259
avzvrgscore -0.1013257  0.09878296  -0.1592273
avzvgtscore  0.3027259 -0.15922725   0.3588957
```

year group 2:

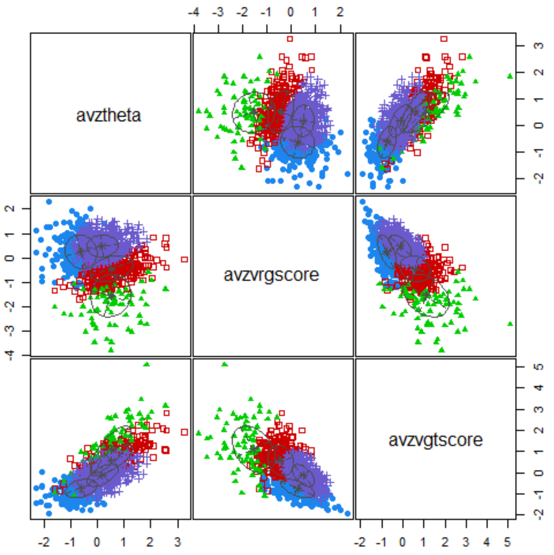

Mclust VVV (ellipsoidal, varying volume, shape, and orientation) model with 4 components:

```
 log-likelihood    n df       BIC       ICL
      -2866.858 1017 39 -6003.775 -6651.111

Clustering table:
  1   2   3   4 
221 243  72 481 

Mixing probabilities:
         1          2          3          4 
0.22119831 0.24591102 0.09710938 0.43578128 

Means:
                  [,1]       [,2]       [,3]       [,4]
avztheta    -0.6207975  0.5194824  0.4940832  0.1758524
avzvrgscore  0.2471411 -0.4445489 -1.5465528  0.4840598
avzvgtscore -0.7444219  0.5827136  1.1063159 -0.1262522

Variances:
[,,1]
            avztheta avzvrgscore avzvgtscore
avztheta     0.35727047 -0.03710753  0.09732007
avzvrgscore -0.03710753  0.49066762 -0.18360282
avzvgtscore  0.09732007 -0.18360282  0.19489920
[,,2]
            avztheta avzvrgscore avzvgtscore
avztheta    0.6386368  0.13671126  0.43032982
avzvrgscore 0.1367113  0.28873416 -0.06103469
avzvgtscore 0.4303298 -0.06103469  0.51713352
[,,3]
            avztheta avzvrgscore avzvgtscore
```

```
avztheta    0.5723351   0.0617003   0.6298972
avzvrgscore 0.0617003   0.7670338  -0.3320269
avzvgtscore 0.6298972  -0.3320269   1.2146832
[,,4]
            avztheta avzvrgscore avzvgtscore
avztheta    0.3520813   0.0171557   0.1762212
avzvrgscore 0.0171557   0.2002392  -0.1275669
avzvgtscore 0.1762212  -0.1275669   0.2746721
```

For year group 3 we did not compute a clustering.

Year group 4

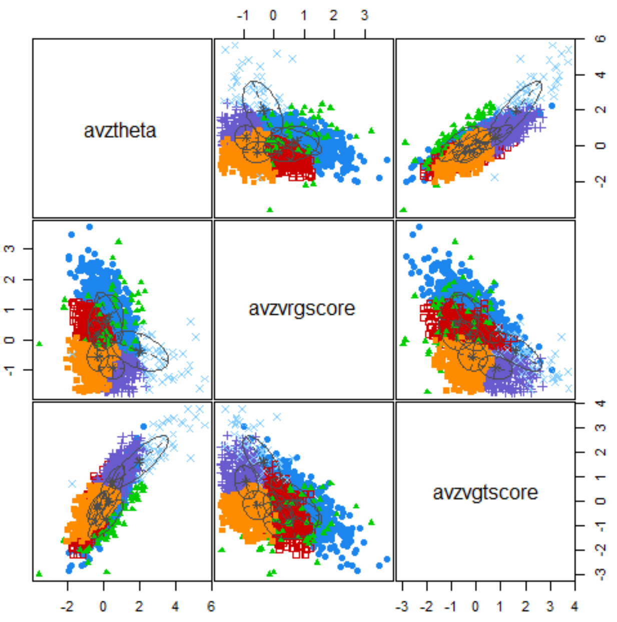

Mclust VVV (ellipsoidal, varying volume, shape, and orientation) model with 6 components:

```
 log-likelihood    n df       BIC       ICL
      -7897.099 2615 59 -16258.47 -18605.38

Clustering table:
  1   2   3   4   5   6
708 875  57 411 518  46

Mixing probabilities:
         1          2          3          4          5          6
0.26300196 0.29461602 0.05050556 0.14683982 0.21381574 0.03122091

Means:
                   [,1]       [,2]       [,3]       [,4]       [,5]      [,6]
avztheta    0.279652903 -0.3454482  0.1169957  0.4717198 -0.3338673  1.9882998
avzvrgscore 0.729848524  0.1366907  0.5690916 -0.9229892 -0.5624563 -0.3764606
avzvgtscore 0.001698588 -0.2930398 -0.7159025  0.7791787 -0.1511236  1.6030333

Variances:
[,,1]
              avztheta avzvrgscore avzvgtscore
avztheta     0.5871565  -0.4096925   0.6343877
avzvrgscore -0.4096925   0.7247223  -0.6420498
avzvgtscore  0.6343877  -0.6420498   0.9323903
[,,2]
```

```
              avztheta avzvrgscore avzvgtscore
avztheta     0.2516808  -0.0966312   0.2346588
avzvrgscore -0.0966312   0.2612957  -0.1680522
avzvgtscore  0.2346588  -0.1680522   0.4221446
[,,3]
             avztheta avzvrgscore avzvgtscore
avztheta    0.9632063  0.16301513  0.60730716
avzvrgscore 0.1630151  0.83875162  0.03204615
avzvgtscore 0.6073072  0.03204615  0.64917015
[,,4]
                avztheta  avzvrgscore avzvgtscore
avztheta     0.474485257 -0.009105009  0.36646638
avzvrgscore -0.009105009  0.147343150 -0.04920676
avzvgtscore  0.366466378 -0.049206761  0.46772295
[,,5]
               avztheta  avzvrgscore avzvgtscore
avztheta    0.350377518  0.005313184   0.2470563
avzvrgscore 0.005313184  0.225362555  -0.0255487
avzvgtscore 0.247056279 -0.025548702   0.3448456
[,,6]
              avztheta avzvrgscore avzvgtscore
avztheta     2.6089225  -0.4823371   1.4454391
avzvrgscore -0.4823371   0.4295942  -0.5132179
avzvgtscore  1.4454391  -0.5132179   1.1386466
```

Year group 5

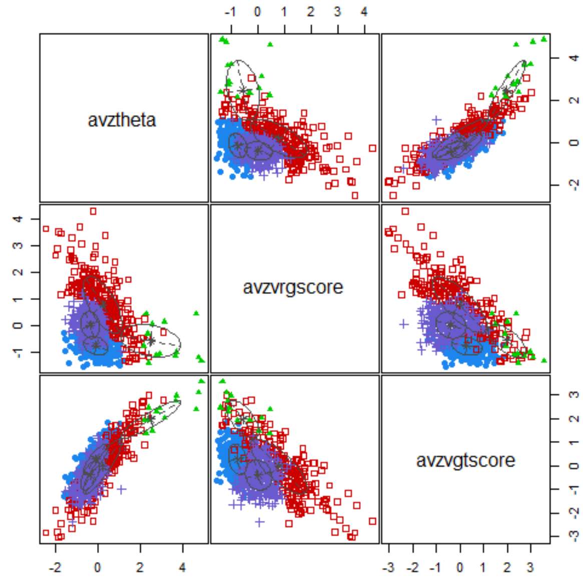


```
----------------------------------------------------
Gaussian finite mixture model fitted by EM algorithm
----------------------------------------------------

Mclust VVV (ellipsoidal, varying volume, shape, and orientation) model with 4 components:

 log-likelihood   n df       BIC       ICL
      -2368.788 800 39 -4998.276 -5410.688

Clustering table:
  1   2   3   4
236 231  13 320

Mixing probabilities:
         1          2          3          4
0.27664317 0.31445831 0.02356288 0.38533563
```

```
Means:
                [,1]        [,2]       [,3]         [,4]
avztheta    -0.1100598  0.21653325  2.4693598 -0.369798029
avzvrgscore -0.7787261  0.77303299 -0.5824511  0.007598848
avzvgtscore  0.2794717 -0.01301062  1.9731644 -0.389535444

Variances:
[,,1]
             avztheta avzvrgscore avzvgtscore
avztheta     0.32173529 -0.05358644  0.29103163
avzvrgscore -0.05358644  0.12303145 -0.06050574
avzvgtscore  0.29103163 -0.06050574  0.41874566
[,,2]
            avztheta avzvrgscore avzvgtscore
avztheta     0.8178126  -0.6284330   0.9377301
avzvrgscore -0.6284330   1.1469768  -0.9801039
avzvgtscore  0.9377301  -0.9801039   1.3714847
[,,3]
            avztheta avzvrgscore avzvgtscore
avztheta     1.9734126  -0.2243545   1.0108622
avzvrgscore -0.2243545   0.3783050  -0.2997795
avzvgtscore  1.0108622  -0.2997795   0.6358473
[,,4]
             avztheta avzvrgscore avzvgtscore
avztheta     0.19200952 -0.03042418   0.1687340
avzvrgscore -0.03042418  0.25807641  -0.0474084
avzvgtscore  0.16873398 -0.04740840   0.4088362
```

Step 4: Additional sensitivity analyses.

Although the bootstrapping method for determining the number of clusters, already constitutes a sensitivity analysis on that aspect, here we perform a number of additional analyses.

Sensitivity to randomness: we repeated the clustering for each year group 100 times, using different random seeds. This did not change the model fit parameters (loglikelihood, bic and icl) measurably in the clusterings for year groups 1, 2 and 5.

For year group 4, there were some fluctuations in the cluster fit parameters:

- The loglikelihood varied between -7911.816 and -7887.377, average -7894.993, where the model we used had a likelihood of -7897.099, close to the average.
- The BIC varied between -16287.9 and -16239.03, average -16254.26, where the model we used had a likelihood of -16258.47, again, close to the average.
- The ILC varied between -19439.18 and -18198.42, average -18715.29, where the model we used had a likelihood of -18605.38, also, close to the average.

So, the model used had average fit parameters when regarding influence of randomness in the clustering algorithm.

Sensitivity to number of clusters: Next, we repeated the step, but with one cluster less in the clustering, again using 100 different random seeds.

For year group 1, the parameters were not sensitive to randomness, all three parameters were worse with 3 clusters:

	3 clusters	4 clusters
loglikelihood	-2745.221	-2726.519
BIC	-5692.647	-5724.969
ILC	-6073.154	-6356.94

For year group 2, the parameters were also not sensitive to randomness, all parameters were again worse with 3 clusters:

	3 clusters	4 clusters
Loglikelihood	-2885.573	-2866.858
BIC	-5971.96	-6003.775
ILC	-6569.795	-6651.111

For year group 5, the parameters were also not sensitive to randomness, parameters were again worse with 3 clusters, except for ICL, which is slightly higher with 4 clusters:

	3 clusters	4 clusters
Loglikelihood	-2398.241	-2368.788
BIC	-4990.337	-4998.276
ILC	-5461.119	-5410.688

For year group 4 we also see fluctuations in the model fit parameters with 5 clusters and also with 4 clusters, due to randomness in the algorithm. This allows for a comparison of distributions of the parameters. Below, we display the distribution of these fit parameters. Green indicates the fit parameters for 6 clusters; orange is for 5 clusters and red is for 4 clusters. Although there is some overlap in the distributions, especially with the ICL parameter, in general we see that the green distributions are better: to the right for loglikelihood and to the left for BIC and ICL. This confirms the selection of 6 clusters for year group 4.

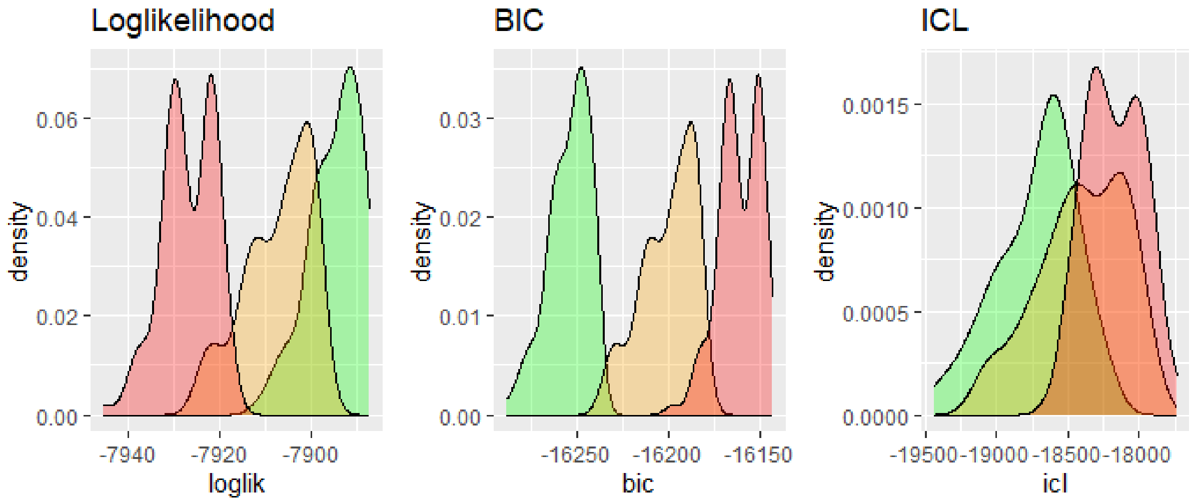

Sensitivity to exclusion of formative exams

In the cluster analysis and subsequent analysis, we excluded data from formative assessments, since the exam conditions fluctuated between medical schools for these formative exams. If formative assessments are included, the number of clusters increases in year group 1 (5 clusters instead of 4) and in year group 3 (2 clusters instead of 1). In year group 2, 4 and 5 the number of clusters is the same. Below we show the clusters with and without formative alongside to compare the structure of the clusters. The coloring of the clusters in the graphs is determined automatically by the Mclust package, so similar clusters can receive another color.

Year group 1: including formative (5 clusters): only summative (4 clusters):

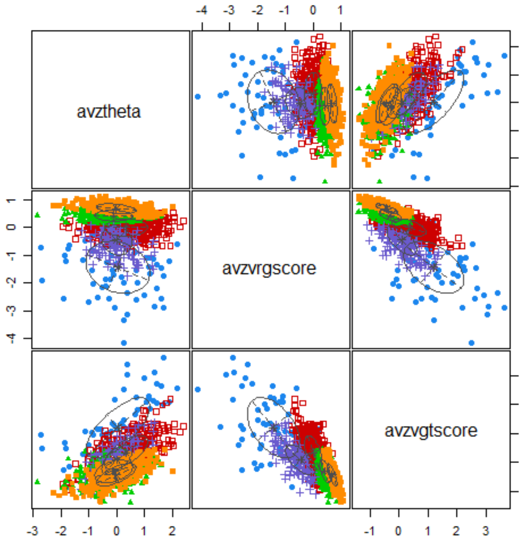

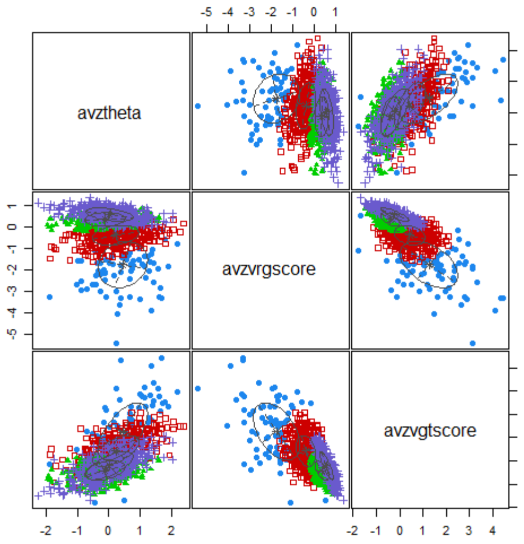

Year group 2: including formative (4 clusters): only summative (4 clusters):

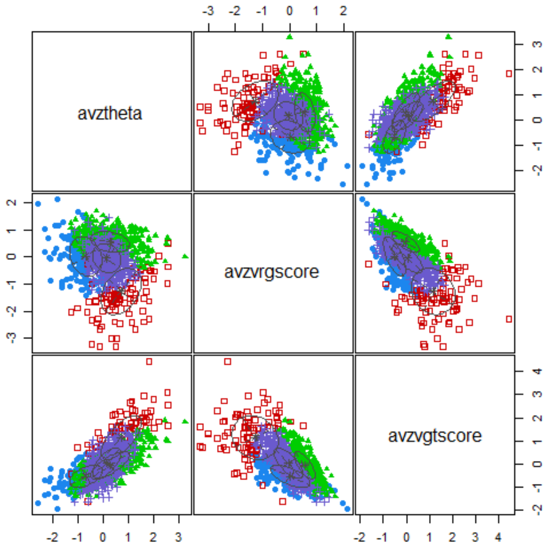

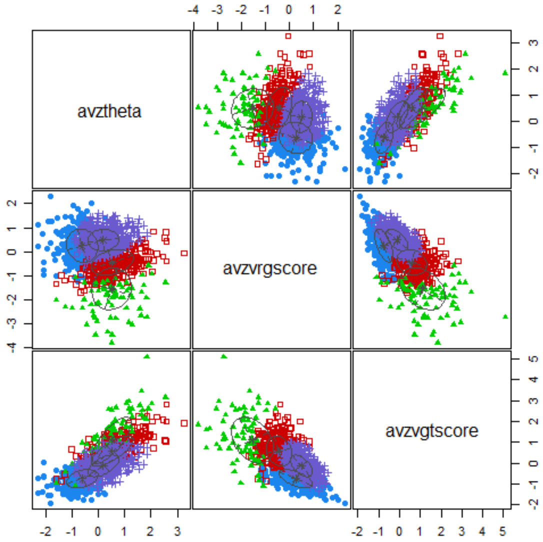

Year group 3: including formative (2 clusters): only summative (no clusters)

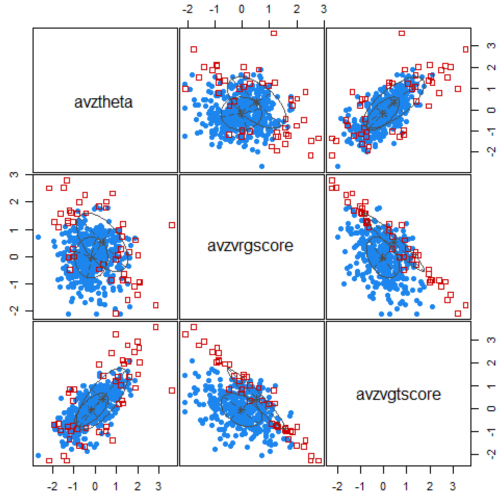

Year group 4: including formative (6 clusters): only summative (6 clusters):

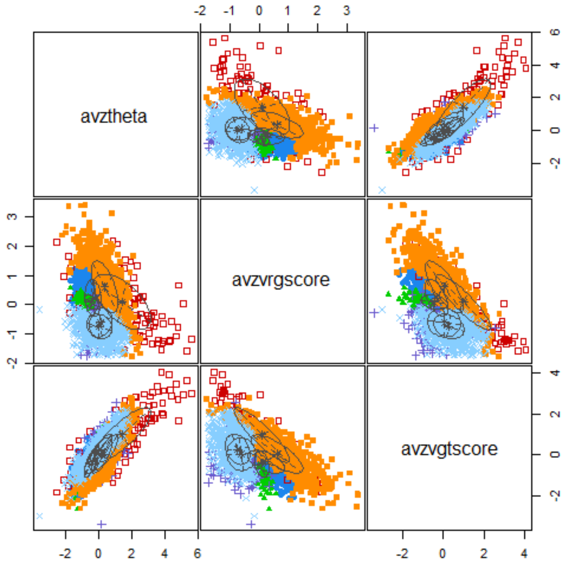

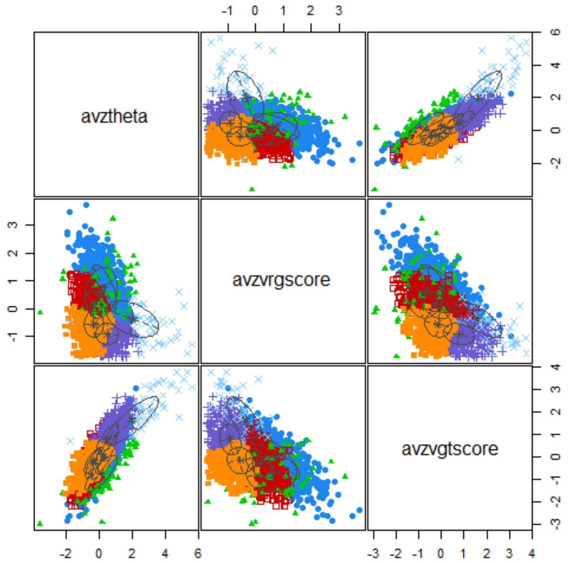

Year group 5: including formative (4 clusters): only summative (4 clusters):

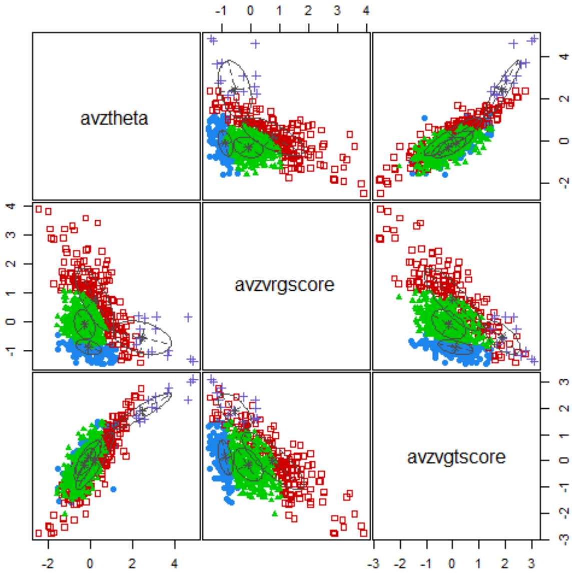

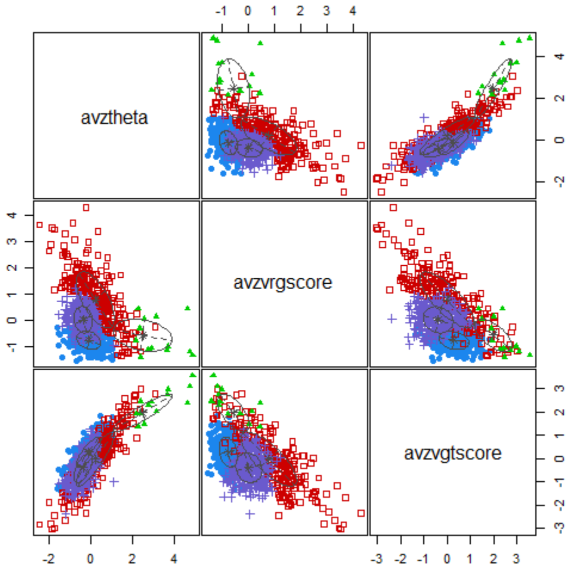

Including formative exams adds data points, and especially in year groups 1 and 3 this leads to a different cluster structure. In year group 1, it mostly means the split of one cluster (cluster 2 in the paper) into two separate clusters. In year group 3, an extremely spread-out cluster appears next to main group. In the other year groups, the cluster structure is comparable.

Effect per cluster

To determine the difference in effect of question-mark use on the CA-PT score, we fitted a linear model per year group, including the cluster-label as covariate in the equation. Effect plots were computing using the effects package [3] in R. Below we provide the fit results, effect plots for the clusters and a visualization of the clusters for reference. Figure titles are in Dutch but should not offer problems for English readers.

Year group 1

```
Call:
lm(formula = avztheta ~ avzvgtscore + avzvrgscore * Cluster3d,
    data = avzlabeled %>% filter(jaargroep == 1))

Residuals:
     Min       1Q   Median       3Q      Max 
-1.95769 -0.32701 -0.00641  0.34518  1.78022 

Coefficients:
                       Estimate Std. Error t value Pr(>|t|)    
(Intercept)             0.42530    0.14874   2.859 0.004328 ** 
avzvgtscore             0.82142    0.03142  26.145  < 2e-16 ***
avzvrgscore             0.56771    0.06686   8.491  < 2e-16 ***
Cluster3d2             -0.19319    0.15518  -1.245 0.213434    
Cluster3d3             -0.61059    0.15696  -3.890 0.000106 ***
Cluster3d4             -0.42951    0.16175  -2.655 0.008042 ** 
avzvrgscore:Cluster3d2  0.28888    0.10368   2.786 0.005426 ** 
avzvrgscore:Cluster3d3  0.69584    0.13268   5.245 1.89e-07 ***
avzvrgscore:Cluster3d4 -0.23750    0.12144  -1.956 0.050764 .  
---
Signif. codes:  0 ‘***’ 0.001 ‘**’ 0.01 ‘*’ 0.05 ‘.’ 0.1 ‘ ’ 1

Residual standard error: 0.5249 on 1058 degrees of freedom
Multiple R-squared:  0.5004,	Adjusted R-squared:  0.4967 
F-statistic: 132.5 on 8 and 1058 DF,  p-value: < 2.2e-16
```

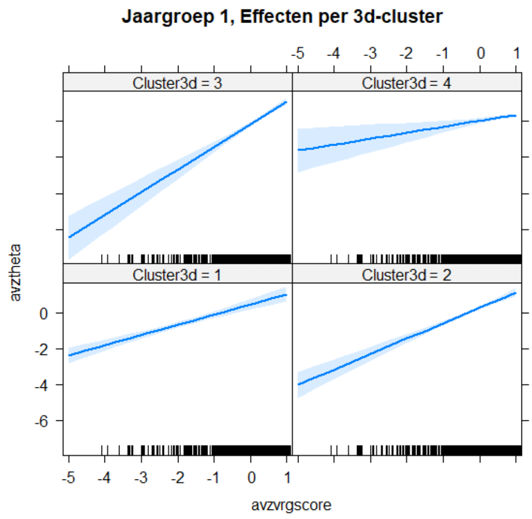

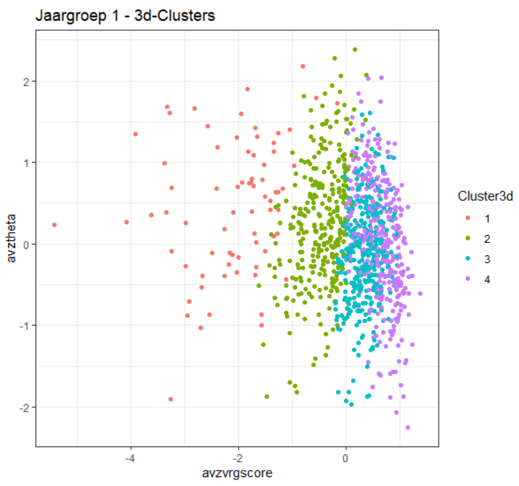

Year group 2

```
Call:
lm(formula = avztheta ~ avzvgtscore + avzvrgscore * Cluster3d,
    data = avzlabeled %>% filter(jaargroep == 2))

Residuals:
     Min       1Q   Median       3Q      Max
-1.80158 -0.30170  0.00126  0.29637  1.32754

Coefficients:
                       Estimate Std. Error t value Pr(>|t|)
(Intercept)            -0.16514    0.03683  -4.484 8.16e-06 ***
avzvgtscore             0.72985    0.02637  27.675  < 2e-16 ***
avzvrgscore             0.16573    0.04346   3.814 0.000145 ***
Cluster3d2              0.73300    0.06971  10.514  < 2e-16 ***
Cluster3d3              0.58383    0.16858   3.463 0.000556 ***
Cluster3d4              0.13674    0.05111   2.675 0.007584 **
avzvrgscore:Cluster3d2  0.69812    0.07778   8.976  < 2e-16 ***
avzvrgscore:Cluster3d3  0.27345    0.08868   3.084 0.002100 **
avzvrgscore:Cluster3d4  0.45798    0.06518   7.027 3.89e-12 ***
---
Signif. codes:  0 ‘***’ 0.001 ‘**’ 0.01 ‘*’ 0.05 ‘.’ 0.1 ‘ ’ 1

Residual standard error: 0.4364 on 1008 degrees of freedom
Multiple R-squared:  0.6958,	Adjusted R-squared:  0.6934
F-statistic: 288.2 on 8 and 1008 DF,  p-value: < 2.2e-16
```

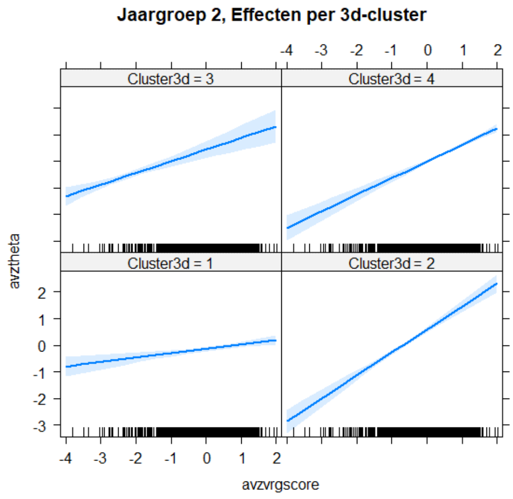

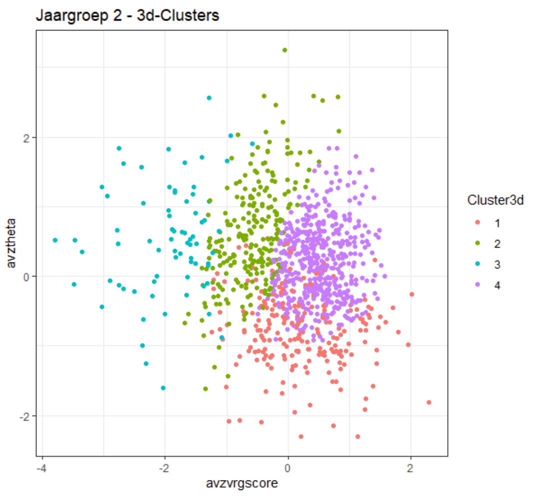

Year group 3

Since there were no clusters in this year group, we only estimate the overall effect.

```
Call:
lm(formula = avztheta ~ avzvgtscore + avzvrgscore, data = avzlabeled %>%
    filter(jaargroep == 3))

Residuals:
     Min       1Q   Median       3Q      Max
-1.78413 -0.36203  0.00889  0.33148  2.71094

Coefficients:
            Estimate Std. Error t value Pr(>|t|)
(Intercept) -0.08526    0.02837  -3.005  0.00281 **
avzvgtscore  0.82463    0.03558  23.180  < 2e-16 ***
avzvrgscore  0.30656    0.03519   8.711  < 2e-16 ***
---
Signif. codes:  0 '***' 0.001 '**' 0.01 '*' 0.05 '.' 0.1 ' ' 1

Residual standard error: 0.5716 on 412 degrees of freedom
Multiple R-squared:  0.5686,	Adjusted R-squared:  0.5665
F-statistic: 271.6 on 2 and 412 DF,  p-value: < 2.2e-16
```

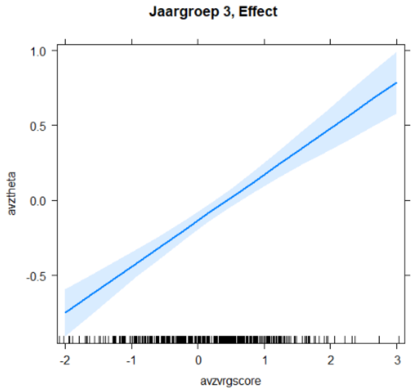

Year group 4

```
Call:
lm(formula = avztheta ~ avzvgtscore + avzvrgscore * Cluster3d,
    data = avzlabeled %>% filter(jaargroep == 4))

Residuals:
    Min      1Q  Median      3Q     Max 
-3.9125 -0.2638  0.0018  0.2489  2.0734 

Coefficients:
                      Estimate Std. Error t value Pr(>|t|)    
(Intercept)            0.38729    0.02571  15.066  < 2e-16 ***
avzvgtscore            0.63578    0.01460  43.551  < 2e-16 ***
avzvrgscore           -0.05692    0.02458  -2.315 0.020664 *  
Cluster3d2            -0.55120    0.03091 -17.834  < 2e-16 ***
Cluster3d3             0.54851    0.06953   7.889 4.45e-15 ***
Cluster3d4            -0.14520    0.06878  -2.111 0.034874 *  
Cluster3d5            -0.66856    0.05132 -13.027  < 2e-16 ***
Cluster3d6             1.38718    0.09473  14.644  < 2e-16 ***
avzvrgscore:Cluster3d2  0.01256    0.04153   0.303 0.762273    
avzvrgscore:Cluster3d3  0.22860    0.05941   3.848 0.000122 ***
avzvrgscore:Cluster3d4  0.27088    0.06652   4.072 4.79e-05 ***
avzvrgscore:Cluster3d5  0.08209    0.05814   1.412 0.158104    
avzvrgscore:Cluster3d6  0.20388    0.10697   1.906 0.056763 .  
---
Signif. codes:  0 ‘***’ 0.001 ‘**’ 0.01 ‘*’ 0.05 ‘.’ 0.1 ‘ ’ 1

Residual standard error: 0.3987 on 2602 degrees of freedom
Multiple R-squared:  0.7857,	Adjusted R-squared:  0.7848 
F-statistic: 795.2 on 12 and 2602 DF,  p-value: < 2.2e-16
```

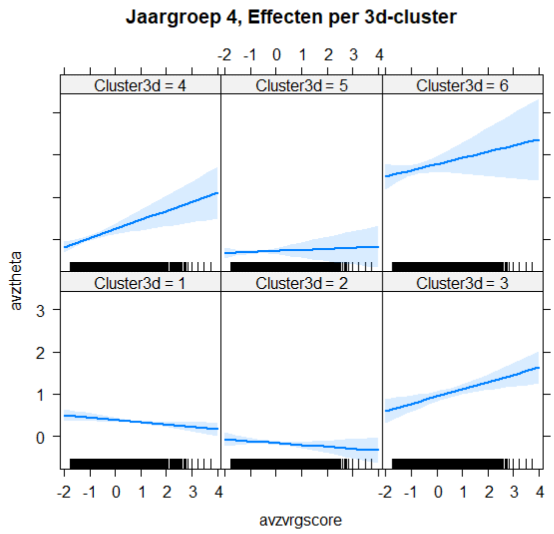

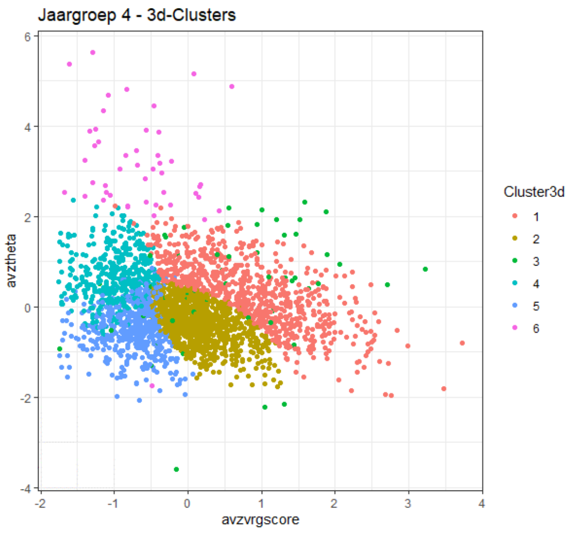

Year group 5

```
Call:
lm(formula = avztheta ~ avzvgtscore + avzvrgscore * Cluster3d,
    data = avzlabeled %>% filter(jaargroep == 5))

Residuals:
     Min       1Q   Median       3Q      Max 
-1.00882 -0.24045 -0.01852  0.25680  1.63538 

Coefficients:
                       Estimate Std. Error t value Pr(>|t|)    
(Intercept)            -0.52708    0.07460  -7.066 3.51e-12 ***
avzvgtscore             0.58172    0.02086  27.889  < 2e-16 ***
avzvrgscore            -0.28429    0.08388  -3.389 0.000735 ***
Cluster3d2              0.91896    0.08307  11.063  < 2e-16 ***
Cluster3d3              2.44794    0.16662  14.691  < 2e-16 ***
Cluster3d4              0.38988    0.07791   5.004 6.92e-07 ***
avzvrgscore:Cluster3d2  0.17015    0.08738   1.947 0.051854 .  
avzvrgscore:Cluster3d3  0.39202    0.17518   2.238 0.025512 *  
avzvrgscore:Cluster3d4  0.11376    0.09785   1.162 0.245385    
---
Signif. codes:  0 '***' 0.001 '**' 0.01 '*' 0.05 '.' 0.1 ' ' 1

Residual standard error: 0.3688 on 791 degrees of freedom
Multiple R-squared:  0.8019,	Adjusted R-squared:  0.7999 
F-statistic: 400.2 on 8 and 791 DF,  p-value: < 2.2e-16
```

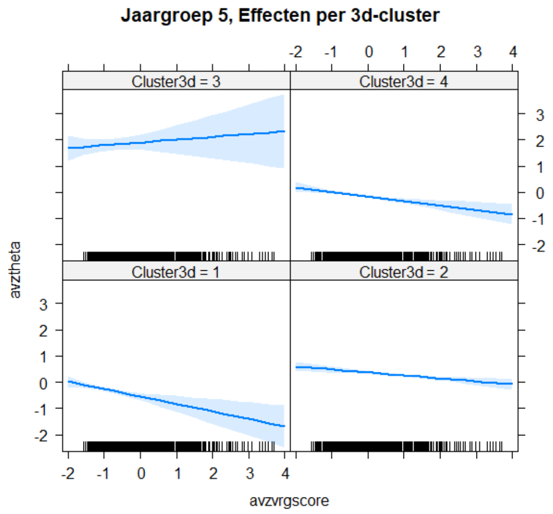

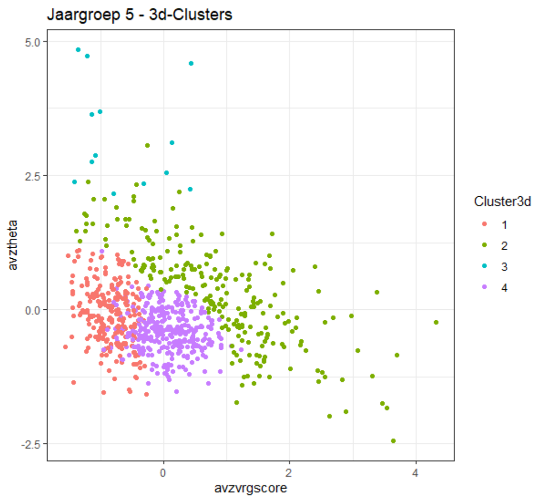

Sensitivity for formative tests

Since we excluded formative tests from our data, below we compare the effect plots for including and excluding formative tests side by side per year group. The cluster numbering differs, so similar clusters in both sets can have different numbers. The goal of the comparison is to judge the overall similarity of the effect distribution over the clusters.

Year group 1: including formative (5 clusters): only summative (4 clusters):

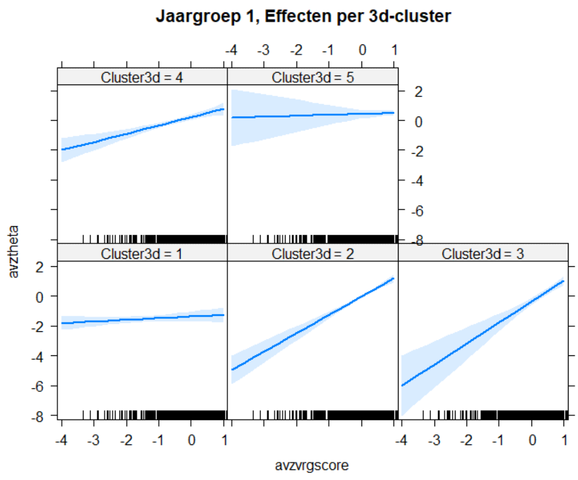

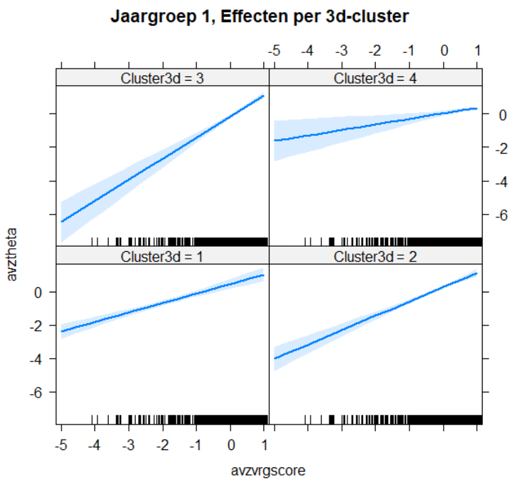

Year group 2: including formative (4 clusters): only summative (4 clusters):

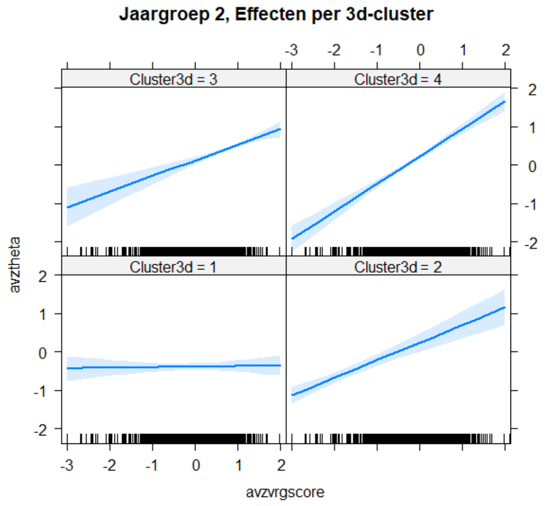

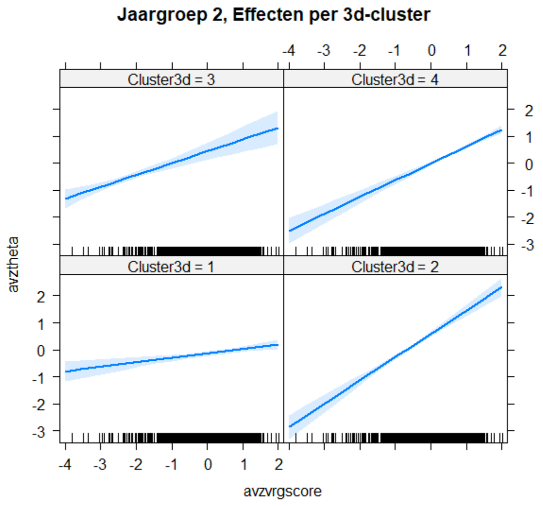

Year group 3: including formative (2 clusters): only summative (1 cluster):

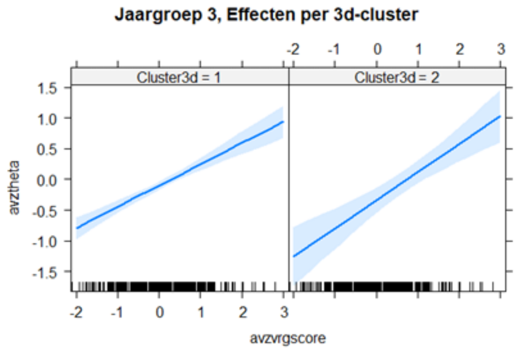

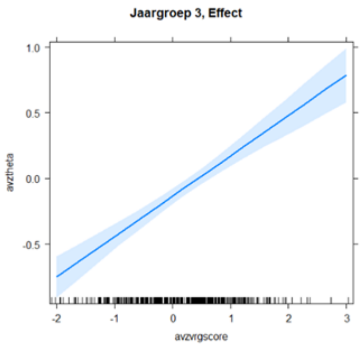

Year group 4: including formative (6 clusters): only summative (6 clusters):

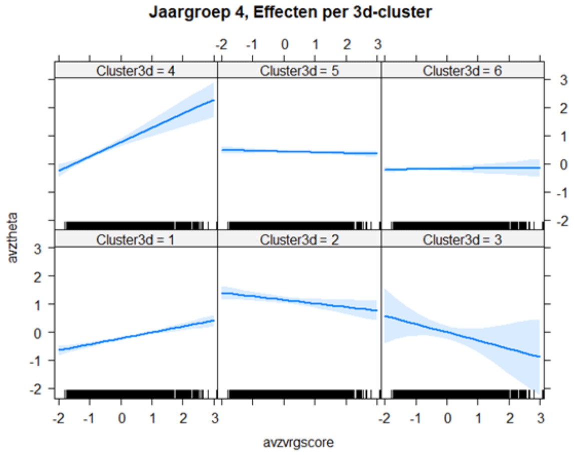

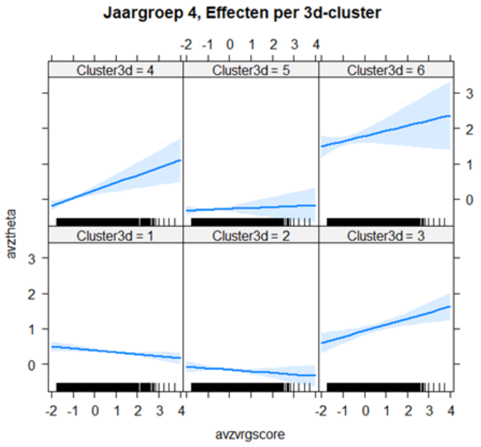

Year group 5: including formative (4 clusters): only summative (4 clusters):

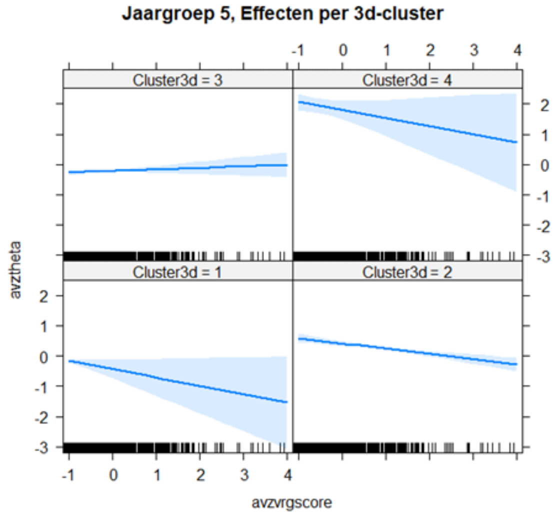

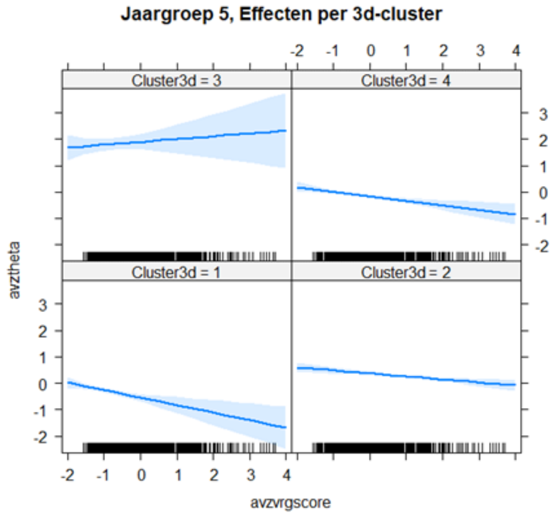

The general patterns in both sets are comparable, we see the same shift from positive effects to negative effects over year groups, with the most diversity in year group 4.

References

[1] Scrucca L, Fraley C, Murphy TB, Raftery AE (2023). “Model-Based Clustering, Classification, and Density Estimation Using mclust in R”. Chapman and Hall/CRC.
ISBN 978-1032234953, doi:10.1201/9781003277965,
https://mclust-org.github.io/book/

[2] R Core Team (2024). “R: A Language and Environment for Statistical Computing.” R Foundation for Statistical Computing, Vienna, Austria. https://www.R-project.org/

[3] John Fox and Sanford Weisberg (2019). An R Companion to Applied Regression, 3rd Edition. Thousand Oaks, CA https://socialsciences.mcmaster.ca/jfox/Books/Companion/index.html
